# Zinc Anode for Mild Aqueous Zinc-Ion Batteries: Challenges, Strategies, and Perspectives

**DOI:** 10.1007/s40820-021-00782-5

**Published:** 2022-01-03

**Authors:** Jinzhang Yang, Bosi Yin, Ying Sun, Hongge Pan, Wenping Sun, Baohua Jia, Siwen Zhang, Tianyi Ma

**Affiliations:** 1 Key Laboratory for Green Synthesis and Preparative Chemistry of Advanced Materials of Liaoning Province, Institute of Clean Energy Chemistry, College of Chemistry, Liaoning University, Shenyang, 110036 People’s Republic of China; 2 Institute of Science and Technology for New Energy, Xi’an Technological University, Xi’an, 710021 People’s Republic of China; 3 State Key Laboratory of Clean Energy Utilization, School of Materials Science and Engineering, Zhejiang University, Hangzhou, 310027 People’s Republic of China; 4 Centre for Translational Atomaterials, Swinburne University of Technology, Hawthorn, VIC 3122 Australia

**Keywords:** Zn-ion batteries, Zn metal anode, Dendrite, Hydrogen evolution, Corrosion

## Abstract

The rapid advance of mild aqueous zinc-ion batteries (ZIBs) is driving the development of the energy storage system market. But the thorny issues of Zn anodes, mainly including dendrite growth, hydrogen evolution, and corrosion, severely reduce the performance of ZIBs. To commercialize ZIBs, researchers must overcome formidable challenges. Research about mild aqueous ZIBs is still developing. Various technical and scientific obstacles to designing Zn anodes with high stripping efficiency and long cycling life have not been resolved. Moreover, the performance of Zn anodes is a complex scientific issue determined by various parameters, most of which are often ignored, failing to achieve the maximum performance of the cell. This review proposes a comprehensive overview of existing Zn anode issues and the corresponding strategies, frontiers, and development trends to deeply comprehend the essence and inner connection of degradation mechanism and performance. First, the formation mechanism of dendrite growth, hydrogen evolution, corrosion, and their influence on the anode are analyzed. Furthermore, various strategies for constructing stable Zn anodes are summarized and discussed in detail from multiple perspectives. These strategies are mainly divided into interface modification, structural anode, alloying anode, intercalation anode, liquid electrolyte, non-liquid electrolyte, separator design, and other strategies. Finally, research directions and prospects are put forward for Zn anodes. This contribution highlights the latest developments and provides new insights into the advanced Zn anode for future research.

**Supplementary Information:**

The online version contains supplementary material available at 10.1007/s40820-021-00782-5.

## Highlights

- This article mainly summarizes the challenges and the latest research progress of highly reversible zinc anodes in mild aqueous zinc-ion batteries and proposes corresponding recommendations.

**Supplementary Information:**

The online version contains supplementary material available at 10.1007/s40820-021-00782-5.

## Graphical abstract

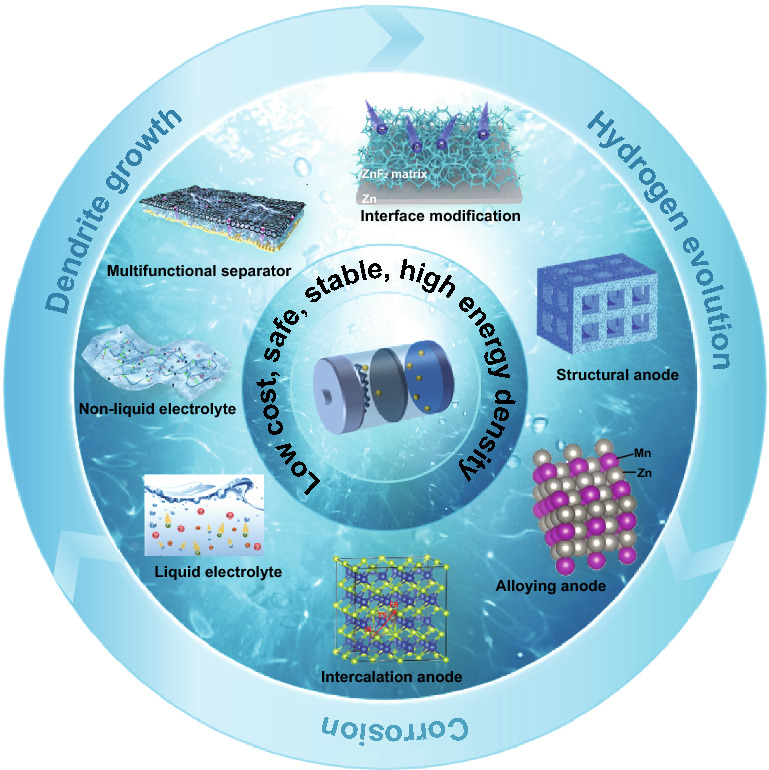

## Introduction

With the sharp increase in energy demand, in response to the global challenges of the increased depletion of traditional fossil energy and associated environmental issue [1–3], many countries and regions have increased their investment in renewable energy such as solar energy, wind energy, and hydropower energy [4–6]. Although there are low- or non-carbon clean energy sources, the generation and conversion of the renewable power supply systems are intermittent, unstable, and uncontrollable, which makes energy storage and transportation difficult [7, 8]. Therefore, as a medium to regulate electricity output and improve the tolerance ability of the power grid to renewable energy, the development of ESS technology is highly essential to enable a clean energy transition [9]. Among the various ESSs, non-aqueous lithium-ion batteries (LIBs) are currently the most widely used rechargeable electrochemical devices [10, 11]. However, owing to flammable organic electrolytes and highly reactive lithium substances, the increasing concerns about the potential safety issues hinder the application of LIBs on a large scale [12–14]. Besides, the high cost and also the low abundance of lithium resources on the earth limited the long-term development of LIBs [15, 16].

Compared with traditional organic electrolytes-based LIBs, aqueous metal-ion batteries have proved promising for large-scale energy storage since the adopted aqueous electrolyte possesses the characteristic of more safety, lower cost, easier processing, and higher ionic conductivity. Currently, various aqueous metal-ion batteries have been developed, such as zinc-ion batteries (ZIBs), sodium-ion batteries (SIBs), potassium-ion batteries (PIBs), aluminum-ion batteries (AIBs), magnesium-ion batteries (MIBs), and calcium-ion batteries (CIBs) [17]. Compared to other active metals, Zn metal can be directly used as an anode due to its proper redox potential (-0.76 V vs. standard hydrogen electrode (SHE)) and excellent Zn/Zn^2+^ reversibility aqueous media. The high natural abundance (approximately 300 times higher than that of lithium) and good resistance to the environment allow Zn to be purchased and processed inexpensively. Very importantly, the Zn anode also has the inherent advantage of high theoretical capacity (820 mAh g^−1^ and 5854 mAh cm^−3^). Therefore, aqueous ZIBs have attracted sufficient attention [18, 19]. According to the pH of electrolytes, aqueous ZIBs can generally be divided into alkaline ZIBs and mild (neutral or mildly acidic) ZIBs. The research of zinc-based batteries traces its history back to the nineteenth century. It was not until the 1980s that Yamamoto et al. first studied rechargeable Zn–MnO_2_ batteries in 2 M ZnSO_4_ electrolyte, which created a precedent for the development of mild aqueous ZIBs [20, 21]. It has been confirmed that the substitution of mild electrolyte for alkaline electrolyte can usually exhibit better reversibility due to many advantages, such as eliminating passivation and alleviating dendrite growth on the anode surface. Thus, these reports about mild aqueous ZIBs have emerged endlessly in recent years.

As an integral part of ZIBs, it is well known that anodes are particularly important to the performance and lifespan of batteries. However, although Zn anodes have inherent advantages, as mentioned above, there are also some thorny problems, which may be devastating to ZIBs. Besides, considerable research efforts have been devoted to the cathode side, such as manganese-based and vanadium-based materials [22, 23], while the research focus on the anode side is still in the primitive stage [24]. Inadequate exploration of Zn anodes leads to a vague understanding of Zn anode failure. It remains a challenge to solve the problems of Zn dendrite growth, hydrogen evolution, and corrosion on the Zn anode side in mild aqueous ZIBs. Based on the previous reports, dendrite growth significantly reduces Zn anode’s capacity and Coulomb efficiency (CE). Large dendrites can even pierce the battery separator and cause a short circuit due to the high mechanical strength and Young’s modulus of Zn. Besides, compared with the traditional alkaline electrolyte, the weakly acidic environment provides a stronger thermodynamic trend for hydrogen evolution. The irreversible consumption of electrolytes during hydrogen evolution and corrosion impairs the long-term cycling performance of ZIBs, and the generated hydrogen increases the risk of battery splitting and electrolyte leakage. Even worse, the mutual reinforcement among these issues causes further deterioration of anode performance [25]. Therefore, in-depth study and optimization of Zn anodes are necessary for ZIBs to move toward practical applications.

To construct highly stable Zn anodes, researchers have proposed various strategies in mild aqueous ZIBs. Considering that it is essential to improve the comprehension of anode challenges and grasp the direction of anode development via recognizing diverse strategies, a detailed summary is necessary. Some previous reviews generally divided these strategies into the following categories: anode matrix modification, electrolyte optimization, and separator design. However, with an in-depth study on Zn anodes, new discoveries and novel modification strategies continue to emerge, and the previous generalizations can no longer keep pace with the latest progress, especially unable to give in-depth and clear explanations for some basic issues. Therefore, this review summarizes recent research from a broad perspective, including more comprehensive basic knowledge and the latest work on Zn anodes. At the same time, based on a large number of published studies, this work elaborates some specific mechanisms in depth, which were mentioned in other reviews but failed to give a detailed explanation, such as how the following facts profoundly affect Zn deposition: the electronic conductivity and Zn affinity of modification layer, Zn alloying, and so on.

**Fig. 1:**
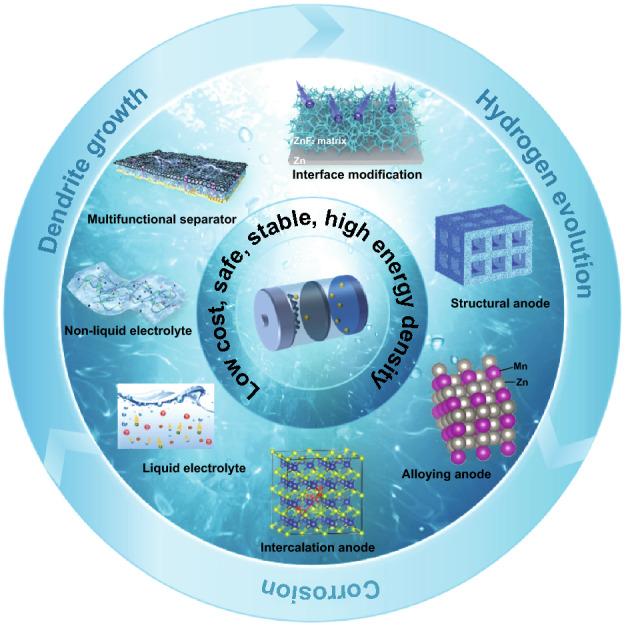
Schematic of strategies to enhance the performance of Zn anodes for mild aqueous ZIBs

This review aims to provide a comprehensive summary of the recent development of Zn anode in mild aqueous ZIBs (as shown in Fig. 1). First, the main challenges, involving dendrite growth, hydrogen evolution, corrosion, and the interaction relationship between them, are systematically analyzed to identify Zn deposition behavior at Zn anode. Then, various latest strategies to enhance the anode performance are categorized and discussed in detail, including interface modification (redistribution of concentration field, redistribution of electric field, and regulation of surface binding energy), structural anode, alloying anode, intercalation anode, liquid electrolyte (weakening of solvation effect, suppression of 2D diffusion, formation of electrostatic shielding layer, and formation of in situ solid electrolyte interphase (SEI) layer), non-liquid electrolyte (solid-state electrolyte, hydrogel electrolyte, and other non-liquid electrolytes), separator design, and other strategies. Finally, by analyzing the latest research achievement, Zn anode’s remaining challenges and perspectives are proposed to produce more reliable aqueous ZIBs with rationally improved performance.

## Challenges of Reversible Zn Anode in Mild Aqueous ZIBs

The possible reactions at the anode/electrolyte interface play significant roles during the energy storage and release of ZIBs. Generally, unlike alkaline systems with zincates as charge carriers, the mild aqueous ZIBs involve the reversible plating/stripping of Zn^2+^ ions on anode surface accompanying the charging/discharging operations. The reaction mechanism of Zn anode can be summarized as

Discharge process:

1 $${\text{Zn}} \to {\text{Zn}}^{{2 + }} + {\mkern 1mu} 2{\text{e}}^{ - }$$

Charge process:

2 $${\text{Zn}}^{{2 + }} + {\mkern 1mu} 2{\text{e}}^{ - } \to {\text{Zn}}$$

**Fig. 2:**
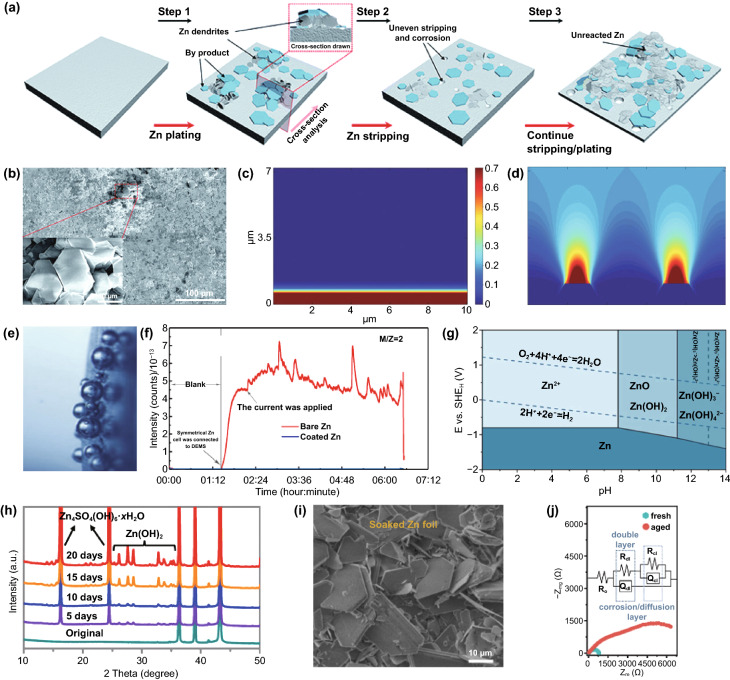
**a** Schematic illustration of the formation of inactive Zn; **b** Top-view SEM image of the Zn electrode after short circuit. Inset: flake-like dendrites [137]. Copyright 2021, Royal Society of Chemistry. Simulation of the diffusion and distribution of Zn ions along the 2D surface of the electrode with the conditions of **c** a flat surface and **d** 2 large dendritic seeds [29]. Copyright 2019, Wiley–VCH. **e** In situ optical microscope images of H_2_ gas evolution during the Zn electrodeposition process at 0.2 mA cm^−2^ [42]. Copyright 2019, Elsevier. **f** Online DEMS data for symmetrical Zn batteries with the bare Zn in 2 M ZnSO_4_ electrolyte, reflecting the hydrogen evolution of the anode during rest and charging/discharging process [43]. Copyright 2019, Royal Society of Chemistry. **g** Pourbaix diagram of ZnSO_4_–H_2_O system at 25 °C [44]. Copyright 2021, American Chemical Society. **h** The in situ XRD patterns of bare Zn immersed in 2 M ZnSO_4_ electrolyte [48]. Copyright 2021, Wiley–VCH. **i** SEM image of Zn foil after soaking in 1 M ZnSO_4_ electrolyte for 7 days [41]. Copyright 2020, Wiley–VCH. **j** Nyquist plots of the fresh and aged Zn electrode. The inset shows the equivalent circuit [49]. Copyright 2021, Wiley–VCH

Simultaneously, given that Zn has a high electrochemical activity and thermodynamical instability in mild aqueous electrolytes, leading to side reactions, there are also anode-related disadvantages, such as dendrite growth, hydrogen evolution and corrosion (Fig. 2a), which will be analyzed as follows.

### Dendrite Growth

Currently, it is generally accepted that Zn dendrite growth is the major problem in mild aqueous ZIBs. Zn dendrites affect battery performance in several ways. On the one hand, due to the loose structure, flake-like Zn dendrites easily fall off the electrode and form “dead Zn,” thus reducing the CE and shortening the battery lifespan (Fig. 2b). On the other hand, the vertical growth of dendrites increases the thickness of the anode, and large dendrites may pierce the separator, causing a short circuit in the batteries. Even worse, faulty batteries may trigger safety incidents such as explosions or fires.

As we all know, Zn dendrites are caused by uneven deposition during the charging process. Specifically, Zn^2+^ ions transfer to the anode surface under the dual effect of the electric field and concentration gradient at the beginning of Zn plating, followed by acquiring electrons and nucleating [26]. Theoretically, nucleation positions should be randomly distributed on an anode surface. But the electrode surface morphology is challenging to be infinitely smooth, and there is a certain degree of roughness. In particular, due to the “tip effect,” compared to other locations, the protrusion with large curvature features higher surface charge density, which stimulates stronger local electric field intensity [27]. Furthermore, even on an infinitely smooth plane, the Zn nucleus formed earlier will act like a “protrusion” to affect the subsequent deposition of Zn^2+^ ions [28]. As a result, driven by the effect of electric field and concentration gradient, Zn^2+^ ions accelerate to accumulate and deposit on the tip (Fig. 2c, d) [29].

It can be known that many factors affect the growth of dendrites in mild aqueous ZIBs, and the connections between them are intricate. It has been proven that the Zn deposition process is affected by electrode polarization, especially concentration polarization. At a specific current density and electrode concentration, Zn^2+^ ions in the electrolyte continuously migrate to the anode reaction interface, where Zn^2+^ ions are consumed. Due to the limited migration speed, the subsequent Zn^2+^ ions cannot be supplemented to the reaction interface in time, resulting in a large concentration gradient perpendicular to the anode surface. The polarization caused by this concentration difference is the concentration polarization. The large concentration polarization is not conducive to the rapid transfer kinetics of Zn^2+^ ions. Besides, concentration polarization can lead to an increase in the overpotential on the anode surface. According to the ultra-thin electric double-layer model on the Zn surface, a large overpotential will seriously disturb the uniformity of the electric field, causes uneven Zn deposition, and destroys the stability and reversibility of the anode [20]. Therefore, the concentration polarization should be as small as possible. In addition, the current density has an important influence on the performance of the Zn anode, which can directly affect the Zn deposition rate. In the diffusion model (Eq. 3), the “Sand’s time τ” is empirically related to the transfer properties of Zn^2+^ ions and electrons as Eq. 3 [30]:

3 $$\tau = \pi D\frac{{eC_{0} \left( {\mu _{a} + \mu _{{Zn^{{2 + }} }} } \right)^{2} }}{{2J\mu _{a} }}$$

where *τ* is the time when Zn dendrites start to grow, and *D* is the diffusion coefficient. *e* is the electronic charge. *C*_0_ is the initial concentration of Zn salt. $\mu _{a}$ and ${\mu }_{{\mathrm{Zn}}^{2+}}$ are the anionic and Zn^2+^ mobility, respectively. *J* is the effective electrode current density. The smaller effective electrode current density (*J*) and larger Zn^2+^ mobility (${\mu }_{{\mathrm{Zn}}^{2+}}$) result in a larger Sand’s time (*τ*), which indicates that the battery has a long lifespan before Zn dendrites grow. This can be explained by the fact that the small current density can lead to a more uniform local current density distribution and a smaller surface electric field distortion [31, 32]. Correspondingly, in a neutral/mildly acidic electrolyte, Yang et al. [29] observed that Zn dendrites gradually appeared and accelerated their growth with the increase in current density. It is proposed that a large current density will increase electrode polarization and cause uneven Zn deposition. On the contrary, a small current density is beneficial to alleviate the dendrite problem. In order to obtain a small current density, a reasonable reduction in the applied current and an increase in the specific surface area of the anode can be considered. At the same time, the capacity (the area capacity corresponding to the area of a symmetric battery or the mass load of the cathode material in an asymmetric battery) has also been found to be another critical factor affecting the growth of Zn dendrites. Large capacity will require more time to complete a single charge process, which leads to more severe dendrite formation [29]. Additionally, perfect metal manufacturing process and polished metal surface/edges can reduce anode defects [33]; the improved Zn affinity of the matrix can lower the nucleation energy barrier [34]; the high operating temperature can lead to increased diffusion coefficient, reduced concentration polarization, large nuclei size, low nucleation density, and compact growth of Zn metal [35, 36]. An appropriately low pH of the electrolyte can enable metal Zn to deposit in a hexagonal structure, instead of the inclined pyramidal structure at high pH [37]. Appropriate external pressure can offset the stress response caused by the intrinsic strain triggered by Zn deposition [38, 39]. By manipulating these factors, the problem of Zn dendrite growth can be alleviated. Based on the above analysis, the appearance of Zn dendrites in neutral or mildly acidic electrolytes largely depends on the battery configuration and charge/discharge protocol.

### Hydrogen Evolution

In addition to Zn deposition, other species may be involved in side reactions on the anode, such as dissolved oxygen in the electrolyte and the electroactive materials of soluble cathodes. But the primary side reaction is the hydrogen evolution reaction (HER) caused by water (Fig. 2e, f) [40]. In fact, due to the influence of various factors such as temperature, applied voltage, electrode surface roughness, and electrolyte concentration, HER is a complicated process, which can be described as follows [41]:

Anode:

4 $${\text{Zn }} \leftrightarrow {\text{ Zn}}^{{2 + }} + {\text{ }}2e^{ - }$$

Cathode hydrogen evolution:

5 $$2{\text{H}}_{2} {\text{O}} + 2{\text{e}}^{ - } \leftrightarrow 2{{\text{OH}}^{ - }} + {{\text{H}}_{2}} \uparrow$$

The hydrogen evolution at the anode occurs during the rest and operation of the battery [42, 43]. Given that the equilibrium potential of Zn^2+^/Zn (−0.76 V vs. SHE) is lower than that of H_2_O/H_2_ (0 V vs. SHE) in the entire pH range (Fig. 2g), the coexistence of Zn and H_2_O is thermodynamically unstable, which means that the two will react spontaneously and release hydrogen [44]. During the plating process, there is a competitive reaction between metal deposition and hydrogen evolution; hydrogen evolution is theoretically preferred over Zn deposition. However, according to the Tafel equation, the Zn mental anode presents a high HER overpotential, which is not conducive to hydrogen evolution kinetics; thus, the rate of HER is limited to a certain degree [45].

The mild aqueous ZIBs suffer from HER, which can irreversibly consume electrodes and electrolytes, thereby reducing battery life and CE. Also, with the gradual accumulation of hydrogen, the battery expands due to the increase in internal pressure until it bursts, ultimately causing electrolyte leakage [46]. In addition, the continuous evolution of hydrogen will cause changes in the local pH of electrolytes (Eq. 5), which is related to the formation of corrosion, exacerbating the negative effect on the battery. It is still an urgent challenge to propose an ideal strategy to solve the hydrogen evolution problem based on the above issues.

### Corrosion

Simultaneously, the corrosion phenomenon accompanying the hydrogen evolution has also attracted long-term attention. Corrosion in mild systems is inseparable from HER, so electrochemical corrosion is also a thorny problem. Specifically, many micro-galvanic cells are formed at the phase interface of Zn metal/electrolyte. Zn metal at the corrosion site is dissolved due to the loss of electrons, and H_2_O in the neutral solution obtains electrons to generate hydrogen and OH^−^ [47]. It has been confirmed that the continuously accumulated OH^−^ will further react with the electrolyte to form by-products on the anode surface. However, different types of electrolytes and operating environments may have different by-products. As we all know, Zn(OH)_2_ and Zn_4_SO_4_(OH)_6_·xH_2_O with a hexagonal structure can be formed in ZnSO_4_ electrolyte (Fig. 2h), and the main reactions involved are as follows [48]:

6 $$4{\rm{Zn}}^{{2^{ + } }} + 6{\rm{OH}}^{ - } + {\rm{SO}}_{4}^{{2 - }} + x{\rm{H}}_{2} {\rm{O}} \leftrightarrow {\rm{Zn}}_{4} {\rm{SO}}_{4} \left( {{\rm{OH}}} \right)_{6} {\rm{ }} \cdot x{\rm{H}}_{2} {\rm{O}}$$

Similarly, other mild electrolytes also participate in Zn corrosion and form by-products, such as TFSI-based complexes which can be formed in Zn(TFSI)_2_ electrolyte. Unfortunately, due to the loose structure of these by-products, they cannot act as a SEI layer to escape from the corrosion reaction (Fig. 2i) [41]. Moreover, the increased interphase impedance caused by the by-product layer restricts the diffusion of electrons/ions, which triggers a high energy barrier for Zn deposition. It severely reduces the performance of the battery (Fig. 2j) [49].

It is worth noting that dendrite growth, hydrogen evolution, and corrosion are inseparable. The three promote each other. The loose and porous Zn dendrites increase the contact area between the electrode and electrolyte, which provides more reaction sites and reduces the current density to achieve a low overpotential, leading to accelerated hydrogen evolution and corrosion. The adhesion of hydrogen bubbles on the anode surface can hinder the nucleation of Zn, resulting in an increased overpotential and uneven Zn deposition. At the same time, the accumulation of OH^−^ anions caused by HER accelerates the corrosion process. The rough Zn surface triggered by corrosion may also further aggravate dendrite formation. The by-product layer with large curvature and irregularity also increases the contact area, accelerating the HER. Therefore, a specific anode modification strategy is usually beneficial to alleviate the three problems simultaneously.

## Design and Optimization of High-performance Zn Anode in Mild Aqueous ZIBs

According to the above analysis, the anode problems, including dendrite growth, hydrogen evolution, and corrosion, hinder the commercialization of mild aqueous ZIBs. It is imperative to explore and develop efficient and stable Zn anode protection strategies. The mild aqueous ZIBs are advancing rapidly; especially in recent years, numerous novel and unique research ideas and achievements continue to emerge. This section summarizes and discusses recent developments from multiple perspectives, including interface modification, structural anode, alloying anode, intercalation anode, liquid electrolyte, non-liquid electrolyte, separator design, and other strategies.

### Interface Modification

In the process of Zn^2+^ ion diffusion, reduction, nucleation, and Zn crystal growth, considering that dendrite growth and HER mainly occur at the anode/electrolyte interface, it seems a direct and effective strategy to construct a surface modification layer. The modified layer usually plays multiple roles, and this section focuses on redistribution of concentration field, redistribution of the electric field, and regulation of surface binding energy. These, respectively, directly manipulate the migration behavior of ions and molecules on the electrolyte side, the electron distribution on the anode side, and the nucleation barrier at the reaction interface to guide uniform Zn nucleation and growth and inhibit side reactions.

#### Redistribution of Concentration Field

The redistribution of concentration field mainly adjusts the spatial distribution and migration behavior of Zn^2+^ ions, H_2_O molecules, hydrated Zn^2+^ ions, and anions at the anode reaction interface, thereby improving subsequent Zn deposition and inhibiting side reactions. As mentioned earlier, restricting the migration behavior of Zn^2+^ ions during the deposition process has a profound impact on dendrite growth. Besides, it is already known that the participation of H_2_O molecules in the anode interface reaction will severely reduce the electrode performance. Specifically, in the electrolyte containing a large amount of free water, Zn^2+^ ion (0.74 Å) can be solvated to form a bulky hydrated structure [Zn(H_2_O)_6_]^2+^ (4.3 Å), which is not conducive to the rapid ion transfer [50]. Moreover, Zn^2+^ ions surrounded by H_2_O molecules are difficult to effectively contact the reaction interface, causing a low electrochemical reaction activity, and [Zn(H_2_O)_6_]^2+^ must be desolvated to participate in the subsequent reaction. Thus, there is an additional demand for energy to overcome the strong interaction between Zn^2+^ ions and H_2_O solvation sheath. Even worse, the free H_2_O molecules transferred from the electrolyte or generated after the desolvation process may participate in side reactions, causing hydrogen evolution and corrosion [51]. Anions in the electrolyte are also related to anode performance. On the one hand, since anions will not be solvated, they have high ion mobility and contribute a lot to ionic conductivity, but this reduces the effective ion conductivity provided by Zn^2+^ ions. On the other hand, anions may participate in the formation of by-products [52]. Based on the above considerations, the regulation mechanisms of modified layers, involving inhibiting the 2D diffusion of Zn^2+^ ions, reducing concentration polarization, reducing the degree of hydration, and restricting H_2_O molecules and anions from entering the anode reaction interface, are conducive to achieving uniform and rapid Zn deposition without hydrogen evolution and corrosion. Therefore, designing a suitable interface modification layer to directly redistribute concentration field is a feasible strategy to enhance the performance of Zn anodes. Generally, a modified layer has multiple interrelated means to achieve these goals. Next, the regulation mechanism of these modified layers will be described separately from the mechanical guidance of confined channels, adsorption guidance of polar groups, and directional electric field guidance of aligned dipoles.

**Fig. 3:**
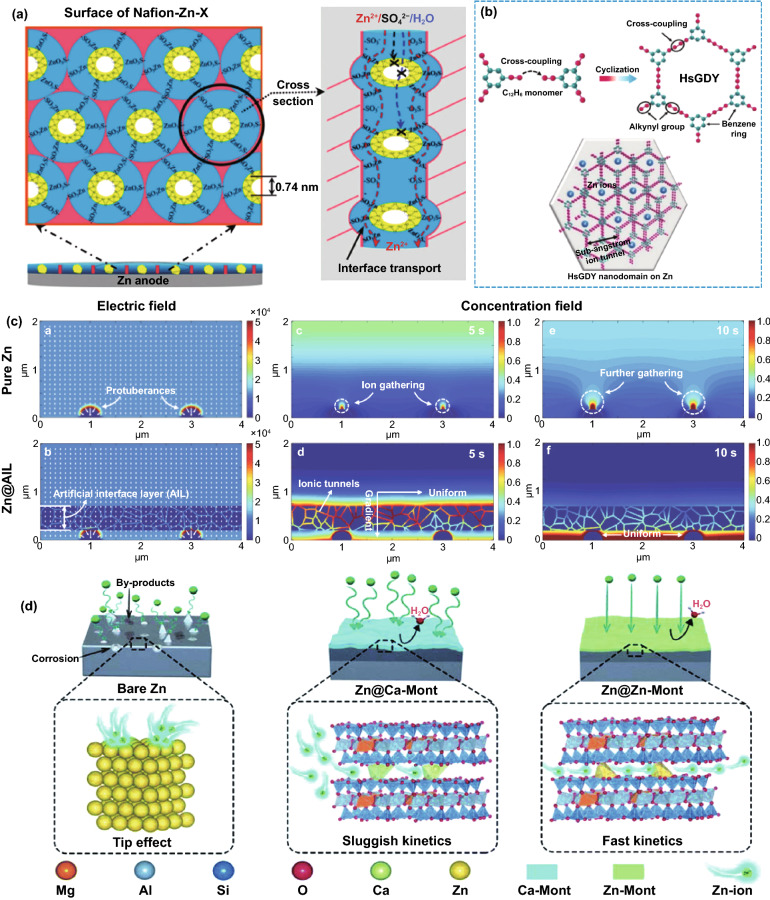
**a** Ion transport mechanisms in Nafion–Zn-X protective layers [54]. Copyright 2020, Wiley–VCH. **b** Schematic illustration of the synthesis of HsGDY and the sub-ångström ion tunnel of HsGDY; **c** Electric and concentration field simulation with protuberances of bare Zn and coated Zn [55]. Copyright 2020, Wiley–VCH. **d** Schematic illustration of the Zn deposition process on bare Zn, Zn@Ca-Mont and Zn@Zn-Mont anodes [58]. Copyright 2021, Elsevier

As a buffer layer that separates the active Zn from the bulk electrolyte, especially electronic insulation properties, the concentration field can generally be mechanically adjusted by constructing the confined channel, such as porous or layered structure. According to the size-selective exclusion effect, to enable the stable migration of Zn^2+^ ions and block other larger-sized molecules and ions, the confined channel structure should be well organized and have an appropriate channel size [53]. For instance, the Nafion–Zn-X modified layer formed by the complexation of Nafion and Zn-X zeolite (Fig. 3a) [54] or the hydrogen-substituted graphodiyne (HsGDY) layer constructed by cross-coupling of C_12_H_6_ monomer (Fig. 3b) [55] and their ion tunnels can only selectively transfer Zn^2+^ ions. In contrast, H_2_O molecules, hydrated Zn^2+^ ions, and SO_4_^2−^ ions with larger sizes cannot pass through the modified layer, so the effective mass transfer is significantly improved. Furthermore, the porous channel structure can serve as a physical barrier to inhibit the 2D diffusion of Zn^2+^ ions. Regardless of the initial uneven electric field on the anode surface, abundant sub-nanometer ion tunnels of HsGDY affect the migration path of Zn^2+^ ions (Fig. 3b): Since Zn^2+^ ions cannot move between tunnels, they can only be transferred along the tunnels and then nucleate and grow on the corresponding anode surface under the tunnels, rather than a thermodynamically favorable adsorption site with a low energy barrier. The inhibited 2D diffusion of Zn^2+^ ions promotes the formation of a uniform Zn^2+^ ion concentration field along with the HsGDY–Zn interface. The simulation of the concentration field well demonstrated the ion redistribution effect of the ion tunnel (Fig. 3c). It has been proved that the channel structure’s high porosity and small pore size are more conducive to accelerating the mass transfer capacity and forming a uniform Zn nucleus. The broadly and densely distributed pores ensure universal and uniform initial nucleation sites on the Zn surface. For some inorganic metal-based compounds, such as nanoporous CaCO_3_ [56], nanoporous SiO_2_ [56], kaolin [57], Zn-based montmorillonite [58, 59], and Mg–Al-layered double hydroxide (LDH) [60], they are also chemically inert and electronically insulating; therefore, the concentration field redistribution in these modified layers is mainly through their confined channel (Fig. 3d), thereby inhibiting dendrites, hydrogen evolution, and corrosion. Obviously, due to the rigid structure of these materials, the lack of mechanical flexibility is not conducive to adapting to changes in the volume of the negative electrode, resulting in limited enhancement of battery performance.

**Fig. 4:**
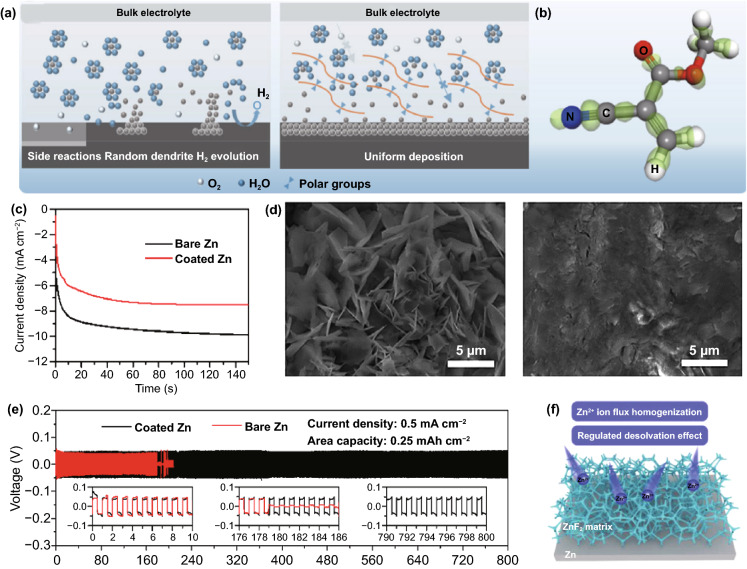
**a** Schematic diagram of the mechanism of cyanoacrylate for suppressing Zn dendrite; **b** The illustrations of electronic cloud distribution of cyanoacrylate monomer; **c** CAs of bare Zn and coated Zn at a 150 mV overpotential; **d** Morphology of bare Zn foil and 502-decorated Zn foil obtained from symmetric Zn cells after Zn stripping/plating for 100 cycles at 0.5 mA cm^−2^ for 0.25 mAh cm^−2^; **e** long-term cycling stability for the symmetrical cells at 0.5 mA cm^−2^ for 0.25 mAh cm^−2^ with the inset showing detailed voltage profile [62]. Copyright 2020, Elsevier. **f** Schematic illustration of the Zn@ZnF_2_ electrode [67]. Copyright 2020, Wiley–VCH

In addition to constructing intuitive confined channels to mechanically change the concentration field, many organic materials can directly and selectively manipulate the migration of Zn^2+^ ions to redistribute concentration field with the assistance of their specific polar groups (Fig. 4a), such as the amide group in polyamide (PA) [43], the poly(vinyl alcohol) group in poly(vinyl butyral) (PVB) [41], the carbonyl group in polyimide [49], the amide group and pyrrolidone group in polyacrylamide (PAM)/polyvinylpyrrolidone (PVP) [61], and the cyano group in cyanoacrylate [62] or polyacrylonitrile (PAN) [63]. These polar groups can donate electron pairs, which can guide coordination adsorption through strong interaction with Zn^2+^ ions (Fig. 4b). Due to the lower energy barrier, Zn^2+^ ions are transferred along the long organic chain containing polar groups, rather than freely diffused, and a large number of long chains create denser and more uniform nucleation sites for Zn deposition, which can be sensitively reflected by the variation in current versus time at a constant potential during the deposition process (Fig. 4c) [62]. At the same time, considering the electrostatic repulsion, the electronegative polar groups can block a certain amount of anion ions. It means that Zn^2+^ ions have a stronger driving force to gather and migrate in the modified layer than other ions or molecules. The priority and selectivity promote the rapid transfer of Zn^2+^ ions and the decrease in the concentration gradient, which can significantly reduce concentration polarization. As a result, stable Zn deposition and stripping can be achieved (Fig. 4d, e). Furthermore, polar groups can change the solvated structure of [Zn(H_2_O)_6_]^2+^ ions to lower desolvation energy. On the one hand, owing to the strong interaction between polar groups and solvated Zn^2+^ ions, polar groups can replace part or all of the H_2_O molecules; on the other hand, the N, O, and F atoms in some polar groups may fix the H_2_O molecules of the solvent sheath by forming hydrogen bonds, resulting in a destroyed sheath structure and reduced coordination number of Zn^2+^ [64–66]. The enhanced desolvation process can increase the corrosion potential and reduce the corrosion current, indicating less tendency and rate of corrosion and hydrogen evolution. In addition to organic materials, some inorganic materials can also interact strongly with Zn^2+^ ions, which has a similar effect on concentration field adjustment. For example, a three-dimensional (3D) interconnected ZnF_2_ matrix could be obtained by electrodeposition in NH_4_F aqueous solution [67]. Compared to bare Zn mental, the ZnF_2_ matrix exhibits stronger Coulomb attraction for Zn^2+^ ions. Coupled with the specific interconnected porous structure, the Zn^2+^ ion flux becomes more uniform, and the desolvation effect and transfer kinetics are enhanced (Fig. 4f). In addition, the S, O, and P atoms in ZnS, ZnO, and ZnP layers, respectively, also have strong adsorption to Zn^2+^ ions, which have been proven to contribute to the concentration field redistribution effect [68–70].

**Fig. 5:**
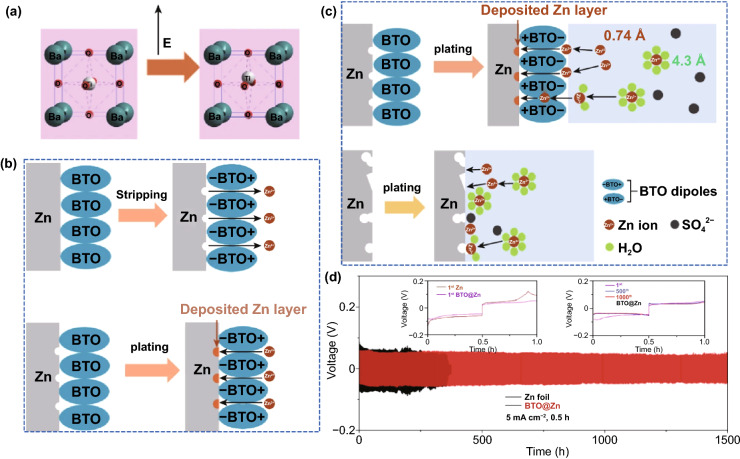
**a** Schematic diagram of the Ti ion migration in the [TiO_6_] octahedral interstitial sites under the external electric field; **b** Schematic of Zn^2+^ ion transport during Zn stripping/plating for BTO@Zn foil; **c** Schematic of the mechanism of Zn^2+^ ion transport at the (top) BTO@Zn/electrolyte and (bottom) Zn anode/electrolyte interface during Zn plating process; **d** Cycling performance of the symmetric cells with Zn and BTO@Zn at 5 mA cm^−2^ with a capacity of 2.5 mAh cm^−2^. The insets reveal the detailed corresponding voltage profiles at various current densities and different cycles [73]

The dielectric material can respond to the external electric field in an inductive manner, producing an electric dipole moment or a change in the electric dipole moment along the direction of the electric field [71]. The directional polarization electric field of the electric dipole moment can adjust the flux of ion charge migration. Precisely, under the applied electric field, the charge in some dielectric materials can move in the microscopic range, resulting in polarization [72]. The additional electric field caused by the polarized charge can manipulate the Zn^2+^ ion migration. It is recently reported that perovskite-type dielectric material BaTiO_3_ (BTO) can be polarized by an external field [73], and the Ti ions in [TiO_6_]^2+^ deviate from the center of the symmetrical position to form an aligned electric dipole (Fig. 5a). Whether in the charging or discharging phase, the excited directional polarization electric field can induce the ordered Zn^2+^ ion migration (Fig. 5b). Impressively, during the plating process, the direction of the polarization electric field can be switched according to the reversal of the external electric field, which simultaneously accelerates the transfer of Zn^2+^ ions, repels anions, and enhances desolvation (Fig. 5c). As a result, the BTO@Zn symmetric cell exhibits decent cycling stability over 1500 h at 5 mA cm^−2^ with the capacity of 2.5 mAh cm^−2^ (Fig. 5d). Additionally, the previously reported ZrO_2_-modified layer can form Maxwell–Wagner polarization due to its high dielectric constant and low conductivity compared to Zn metal [74]. The polarization electric field provides controllable nucleation sites for Zn^2+^ ions and promotes fast ion kinetics, leading to uniform Zn deposition/stripping. Encouragingly, the types of dielectric materials are relatively abundant, which can provide more selectivity for the future design of directional polarization electric fields to redistribute concentration field.

#### Redistribution of Electric Field

In addition to directly changing the flux of molecules and ions on the electrolyte side at the electrolyte/anode interface, directly regulating the electron distribution on the anode side can also manipulate the Zn deposition behavior. The redistribution of electrons can change the distribution of the electric field, mainly homogenizing the local electric field or enlarging the local electric field.

**Fig. 6:**
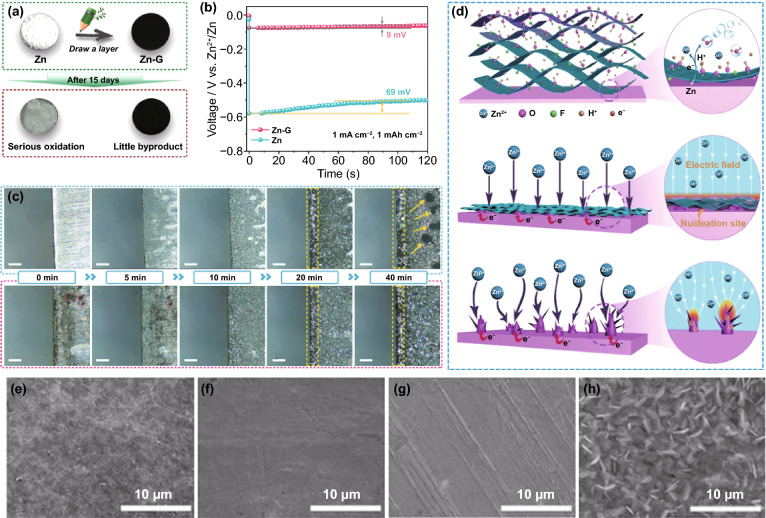
**a** Schematic illustration of the modification process and the stability in 2 M ZnSO_4_ electrolyte of Zn and graphite-coated Zn anode; **b** The voltage–time curves of Zn and Zn–G symmetric cells at 1.5 mA cm^−2^; **c** In situ optical microscope photographs of (top) Zn and (bottom) Zn–G electrodes observed by symmetric transparent cells under various deposition times [78]. Copyright 2020, Wiley–VCH. **d** Illustration of (top) synchronously reducing and assembling MXene layer on the Zn foil surface; Illustration of Zn plating behavior of (middle) MXene-coated Zn, and (bottom) pure Zn; SEM images of MZn-60 and pure Zn **e, g** before cycling, and **f, h** after 100 cycles at 3 mA cm^−2^ [81]. Copyright 2020, Wiley–VCH

The deposition behavior of Zn^2+^ ions dominated by the electric field is greatly affected by the 2D diffusion around the nucleation site. According to the fundamentals of the tip effect, the uneven electric field distribution is caused by the larger local surface charge density. Therefore, the local electric field strength is closely related to the local current density. It can be known from Eq. 3 that while increasing the anode current density in pursuit of a faster battery charging rate, the local current density at the Zn deposition site should be reduced to inhibit the growth of dendrites [30]. Under the premise of a constant applied current, the modified conductive layer with a large specific surface area, such as graphene oxide (GO) [75], reduced graphene oxide (rGO) [76, 77], graphite [78], carbon nanotubes (CNT) [79], and other carbon-based materials, can distribute a part of the electronic charge of the anode, thereby having high electrochemical activity. This provides more selective nucleation sites for Zn deposition instead of converging only in the initial few hot spots for charge transfer. Thus, the local current density is significantly reduced, corresponding to a more uniform local electric field [80]. A novel and simple strategy for constructing a graphite functional interface was proposed as a proof of concept. The graphite layer was painted directly on the anode surface with the assistance of ordinary pencils (Fig. 6a) [78]. Through electrochemical tests, the nucleation overpotential (NOP) value (69 mV) of pure Zn anode was much higher than that of graphite-coated Zn anode (9 mV) (Fig. 6b). This meant that the high conductivity and large surface area of the graphite layer enabled the anode with a lower local current density, which corresponds to the characteristics of smaller Zn nuclei formation and no dendrites. This result was also proved by observing the transparent symmetrical battery through an optical microscope: Some large dendrites (dark spots marked by yellow arrows) were formed on the bare Zn surface within 40 min plating, while the graphite-coated Zn anode had a flat and smooth surface (Fig. 6c). Apart from carbon-based materials, 2D-structured MXene material with metallic conductivity as a modified layer also has a similar effect of homogenizing the local electric field (Fig. 6d) [81]. Even at the large current density, the highly conductive structure network can destroy the tip effect inside the MXene (Ti_3_C_2_T_x_) layer by eliminating the uneven local electric field. Due to the lack of driving force for dendrite growth, the nucleation and growth of Zn are more uniform (Fig. 6e–h).

**Fig. 7:**
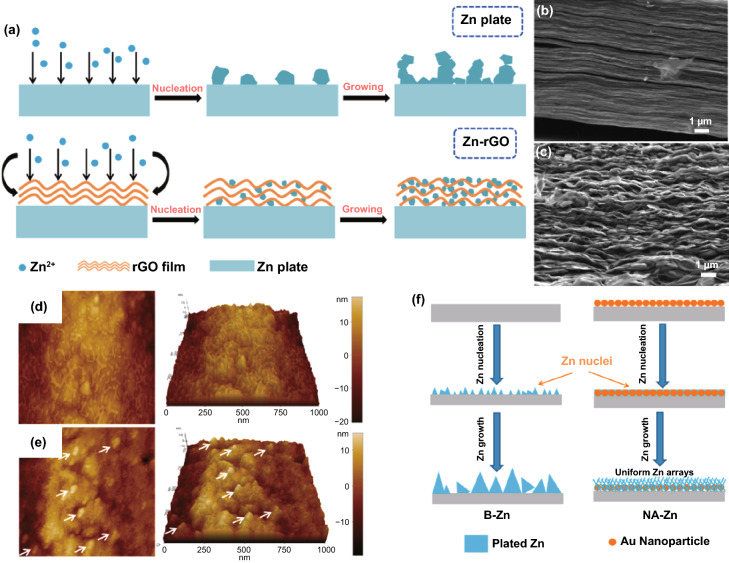
**a** Schematic illustrating the Zn plating behavior of the bare Zn and Zn/rGO anodes; cross-sectional SEM images of rGO film on Zn foil **b** before cycling and **c** after cycling [77]. Copyright 2019, Elsevier. Atomic force microscope images of **d** bare Zn anode and **e** Au-decorated Zn anode; **f** Schematic illustration of the Zn stripping/plating process [84]. Copyright 2019, American Chemical Society

Due to the anode electron redistribution, the possible deposition sites of Zn^2+^ ions include the Zn metal surface, inside, and the surface of the modified conductive layer. The electrical conductivity of some modified layers is lower than that of Zn metal, and there is also generally contact resistance between them, such as ZIF-8 derived carbon [82]. Different electrical conductivity and high contact resistance result in potential change between the modified layer and Zn metal. The potential near the Zn metal surface was low (or negative) enough for Zn^2+^ reduction. Therefore, Zn deposition will preferentially occur at a low potential on the more conductive Zn metal surface, leading to a position-selected, bottom-up Zn deposition process [44]. However, the excellent conductive network has fast electron transfer capability for anodes modified with most carbon-based materials or MXene materials. There is almost no potential change between the modified layer and Zn metal. At a reasonable potential, Zn reduction will occur as long as it contacts the conductive network. Thus, the excellent conductive modified layer is the preferred location for Zn deposition [28]. For example, rGO with a layered structure can generate a stable electric field in which the electrolyte is fully penetrated during the plating process. The Zn^2+^ ions are preferentially distributed uniformly rather than aggregated; Zn is deposited on the inside and surface of the rGO layer (Fig. 7a–c) [76, 77]. It is worth noting that most of the conductive network materials are not structurally complete but have edges and defects on which Zn^2+^ ions will be deposited preferentially. At the same time, the Zn^2+^ ions transferred from the electrolyte will first contact the surface of the modified layer, suggesting that Zn deposited on the surface has a higher priority than the interior. Still, the modified layer surface cannot play the role of homogenizing the electric field. These all mean that the modified layer with a conductive network can only uniform the electric field and suppress dendrites within a certain range. Moreover, due to the increased area contact with the water in the electrolyte, the problem of side reactions at the reaction interface is still troublesome.

Contrary to the strategy of homogenizing the electric field to suppress the 2D diffusion of Zn^2+^ ions, constructing protrusions on the metal surface to enlarge the local electric field to strengthen the 2D diffusion can also suppress dendrites, but the premise is that the protrusions are uniformly and densely distributed on the anode surface [83]. For example, a large number of Au nanoparticles (AU-NP) are constructed on the surface of Zn metal by magnetron sputtering (Fig. 7d, e) [84]. Seed crystals have high curvature and large local electric fields around them, which will preferentially become sites for Zn deposition and growth. The uniform and dense seed crystals enable the deposition of Zn^2+^ ions to be universal instead of growing on several loose tips on the bare Zn surface (Fig. 7f). Actually, during the Zn deposition process, the previously generated Zn nuclei also form a large local electric field to affect subsequent nucleation and growth. Therefore, adjusting the initial nucleation site is of great significance for realizing a dendrite-free anode.

#### Regulation of Surface Binding Energy

**Fig. 8:**
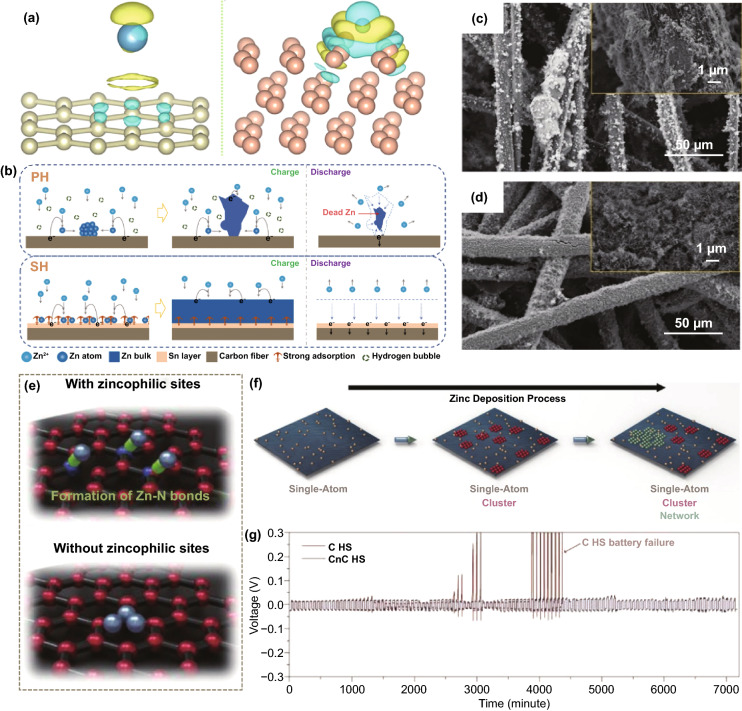
**a** Interfacial charge density of (right) carbon and (left) Sn; **b** Schematic illustration of Zn deposition induction mechanism; SEM images of **c** PH and **d** SH after charging (inset, higher magnification) [87]. Copyright 2019, Wiley–VCH. **e** (top) Spacious Zn nucleation with zincophilic sites and (bottom) dense nucleation on zincophobic surface; **f** Schematic illustration of Zn deposition on surface with zincophilic sites; **g** Galvanostatic cycling performance of symmetric cells with and without zincophilic nitrogen sites [50]. Copyright 2020, Wiley–VCH

According to thermodynamics, Zn nucleation is controlled by the decrease in free energy due to phase transition and the increase in surface energy due to new interfaces. The former is the driving force for nucleation, and the latter leads to a nucleation barrier. Zn preferentially nucleates at the thermodynamically favorable adsorption sites of low energy barriers [85]. The modified material with high Zn affinity has a lower nucleation energy barrier, thus leading to a smaller nucleation overpotential, which is more conducive to Zn nucleation. The Zn affinity is evaluated by comparing the binding energy (calculated by density functional theory) of the Zn atoms attached to the modified layer and the Zn metal surface, and the larger one (more negative) has a higher Zn affinity [86]. It is reported that some metals have high Zn affinity, such as Sn, In, Ag, and Cu; they can serve as heterogeneous seeds to induce Zn nucleation and growth [87–90]. Compared with Zn metal or carbon, a well-organized Sn layer has a uniform and densely distributed zincophilic sites on the surface [87]. During the Zn nucleation process, Zn has stronger electronic interactions with these zincophilic sites (Fig. 8a), and thus, Zn preferentially nucleates uniformly at these sites. The uniformly distributed Zn nuclei also guide the subsequent uniform Zn deposition (Fig. 8b). Therefore, the zincophilic layer can significantly inhibit dendrite growth (Fig. 8c, d). Besides, it was previously proposed that the Li affinity of carbon materials can be enhanced by heteroatom doping, which can be explained by electronegativity, local dipole, and charge transfer. Similarly, the introduction of N-containing sites significantly changes the Zn affinity of graphene [50]. Widely distributed pyridine sites induce the spacious distribution of the initial Zn nuclei by forming Zn–N bonds. Lateral Zn growth leads to nearby connections to form Zn clusters, resulting in uniform Zn deposition (Fig. 8e–g).

**Fig. 9:**
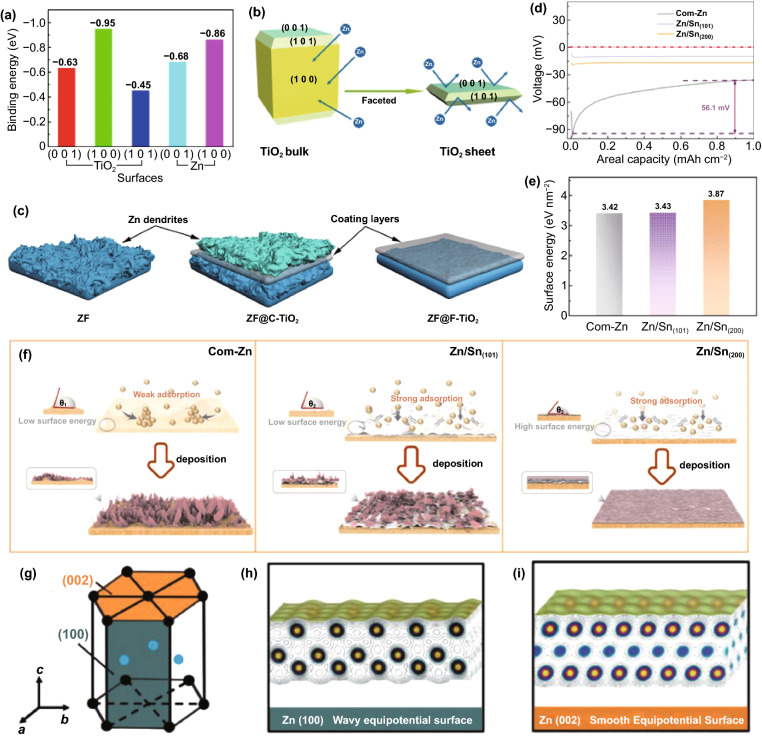
**a** Calculated binding energies of Zn atom with different facets; **b** Schematic illustration of the interaction between Zn and anatase TiO_2_ with different exposed facets; **c** Schematic illustration of the Zn plating process with different coating layers [86]. **d** Voltage profiles of Zn deposition on Com–Zn, Zn/Sn (101), and Zn/Sn (200) at a current density of 1 mA cm^−2^ and a capacity of 1 mAh cm^−2^; **e** The calculated surface energy on different electrodes; **f** Schematic illustration of Zn deposition process on Com–Zn, Zn/Sn (101) and Zn/Sn (200) [91]. Copyright 2020, Wiley–VCH. **g** The structure of metal Zn; Surface atomic arrangement and electron equipotential plane of **h** Zn (100) and **i** Zn (002) [92]. Copyright 2021, Wiley–VCH

Since atoms, molecules, or ions belonging to the non-cubic crystal system have multiple crystal facets, different crystal facets have different binding energies to Zn. Thus, even in the modification layer of the same material, different exposed crystal facets have other effects on Zn nucleation. Consider as-prepared faceted TiO_2_ (F-TiO_2_) nanosheets with exposed (0 0 1) and (1 0 1) facets and commercial TiO_2_ (C-TiO_2_) with exposed (1 0 0) facets, the former with a lower Zn affinity can repel the Zn adsorption, ensuring that Zn^2+^ ions preferentially accumulate and nucleate on the Zn metal under the F-TiO_2_ nanosheets; on the contrary, the high Zn affinity of the latter leads to preferential Zn deposition on the C-TiO_2_ nanosheet surface (Fig. 9a, b) [86]. It is worth noting that the affinity reflects the difficulty of Zn deposition on the substrate. Providing a lower nucleation energy barrier promotes the kinetics of Zn deposition but does not necessarily change the crystal plane orientation of Zn deposition and form a Zn surface texture [91]. As the area capacity increases, it may be beyond the controllable ability of zincophilic sites to Zn deposit; Zn dendrites will still form. The restricted deposition space under the modified layer can further inhibit the large-scale dendrite growth. In contrast, the surface of the modified layer no longer has a constraining effect, and the battery is more likely to be damaged by dendrites (Fig. 9c) [86]. As a similar proof, it has recently been reported that the Sn (1 0 1) surface has a higher Zn affinity than the Sn (2 0 0) surface, and both of them are higher than commercial Zn foils (Fig. 9d). However, the Zn anode with the Sn (1 0 1) surface texture layer still exhibits rough Zn deposition morphology, while the deposition morphology on the Sn (2 0 0) surface texture layer is uniform and smooth. This is derived from the latter’s high average surface energy which may result in particularly strong capillary action, leading to better deposit “wettability” (Fig. 9e, f) [91]. According to the above analysis, the binding energy significantly influences the initial Zn nucleation behavior. At the large area capacity, the modified layer that dominates the Zn deposition with high binding energy does not necessarily play a key role in alleviating the dendrite problem or even may cause negative effects. It is worth mentioning that in the hexagonal close-packed (hcp) Zn lattice, the Zn (0 0 2) crystal surface has a smooth surface and uniform interface charge density, which is not conducive to the formation of dendrites. And such a plane with high atomic coordination has high binding energy, ensuring preferential Zn deposition at these more advantageous sites (Fig. 9g-i). During the deposition process, the surface texture of the Zn (0 0 2) planes can lead to a spontaneous reorientation of Zn crystallites and, finally, maintain the horizontal growth of the Zn (0 0 2) planes through the epitaxial mechanism. At the same time, due to the low electrochemical activity of Zn (0 0 2) planes, corrosion and H_2_ evolution may also be reduced [92, 93]. Therefore, adjusting the surface binding energy by constructing the modified layers and controlling their exposed crystal planes is beneficial for obtaining a stable anode interface and deeply understanding the internal mechanism of binding energy on Zn deposition.

### Structural Anode

Numerous reports have confirmed that the structural design can effectively enhance the overall performance of the ZIBs. Diversified structural design is more and more favored by subsequent research work. The modified layer strategy is mainly based on the Zn deposition of the metal anode plate, which has a limited contact area with the electrolyte. Differently, extending the contact surface to 3D space can significantly improve anode designability. Drastically increased specific surface area is the most crucial feature of structured anodes. Although this may enhance the formation of side reactions, it can effectively increase Zn nucleation sites and reduce local current density. Moreover, sufficient contact with the electrolyte and rapid charge transfer allows for lower polarization. These are conducive to obtaining a stable anode without dendrites. The reported structured anode materials, which can be roughly classified into the metallic matrix material, carbonaceous matrix material, and other matrix materials, exhibit great potential for regulating the Zn deposition behavior.

**Fig. 10:**
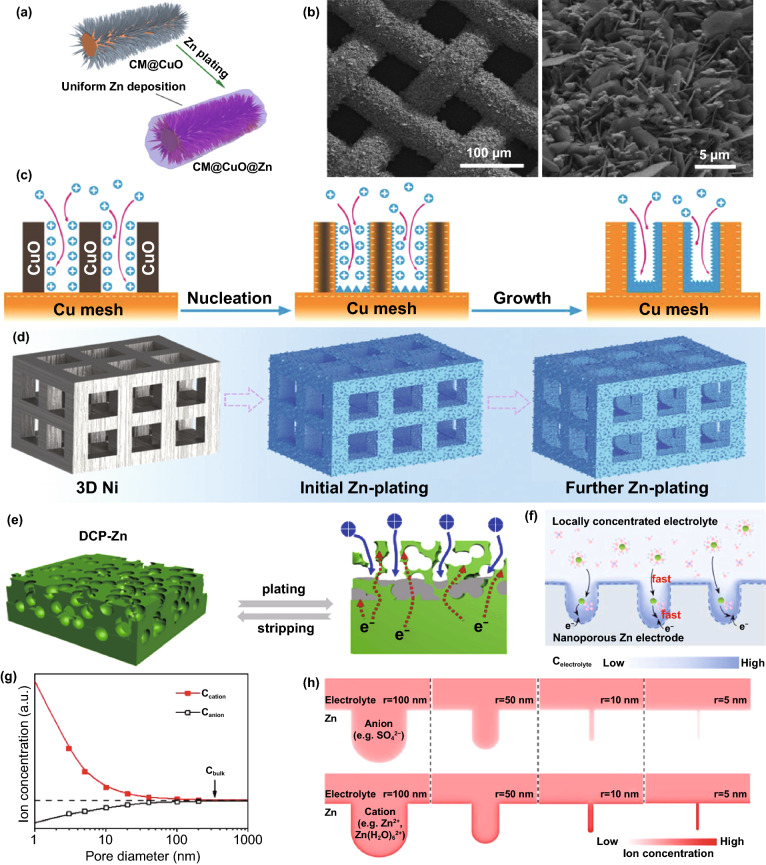
**a** Schematic illustrations of CM@CuO@Zn; **b** SEM images of Zn anode using reduced CM@CuO as the host with the capacity of 5 mAh cm^−2^; **c** Schematic illustrations of the process of Zn deposition on CM@CuO and CM [97]. Copyright 2020, Wiley–VCH. **d** Schematic illustration of Zn deposition on the 3D Ni [98]. Copyright 2020, Wiley–VCH. **e** Stripping/plating performance of DCP-Zn-30 and pristine Zn foil cells with 0.1 mAh cm^−2^ cutoff capacity at 3–10 mA cm^−2^ [99]. Copyright 2020, Elsevier. **f** Nanoporous Zn electrode with interface-localized concentrated electrolyte; **g** The ion concentration at the electric double layer of nanoporous Zn metal with different pore diameters; **h** Surface charge densities of the cations and anions at the interface of ZnSO_4_ electrolyte and nanoporous Zn metal with different pore diameters ranging from 5 to 100 nm [100]. Copyright 2021, Elsevier

To obtain long-lifespan reversible cycle performance, the structured anode that plays a physical structural support role should have good structural stability, which puts forward high requirements for suppressing dendrites. Metal-based structured anodes, especially Cu-based materials (Cu mesh, Cu foam [94, 95], and porous Cu [96]) with excellent Zn affinity, can maintain the morphology of the structure due to their rigid properties. Zn nucleation and growth can be restricted to a specific structure through deliberate design, thereby inhibiting dendrites. Based on the unique structure and function of the Cu mesh, modifying the Cu mesh with CuO nanowires can further expand the specific surface area of the current collector and simultaneously adjust the ion distribution and electric field at the anode (Fig. 10a, b) [97]. Due to its lower nucleation energy barrier, CuO nanowires tend to selectively absorb Zn^2+^ ions and can be reduced to Cu nanowires to form a staggered 3D copper matrix. Zn can be uniformly deposited in the gaps of the nanowires without dendrite formation (Fig. 10c). Similarly, a 3D Ni-Zn anode with a multi-channel lattice structure was fabricated with the help of 3D printing technology (Fig. 10d) [98]. Compared with planar electrodes, 3D Ni-Zn has a larger specific surface area, which redistributes the local electric field and induces the preferential and uniform Zn deposition into the 3D microchannels, thus successfully suppressing dendrites and significantly improving the electrochemical performance of the battery. Besides, Zn itself is also designed as the main body of the 3D structure [99–101]. For example, a 3D porous Zn anode with dual channels, consisting of a continuous cavity and a conductive framework, allows ions and electrons to migrate quickly at the anode interface [99]. The unique cavity structure limits the Zn deposition position, thereby inhibiting dendrite growth (Fig. 10e). Recently, it has been proposed that nanoporous Zn electrodes (npZn) with controllable pore size can be prepared by the alloying–dealloying method. When the pore size is small enough (< 40 nm), the space charge will significantly affect the effective ion concentration of the electrolyte in the pore. Cations (Zn^2+^) are enriched, and anions are reduced so that interface-localized concentrated electrolytes can be achieved (Fig. 10f–h). Zn anode with a nanoporous structure can promote the uniform Zn plating and suppress side reactions [100].

**Fig. 11:**
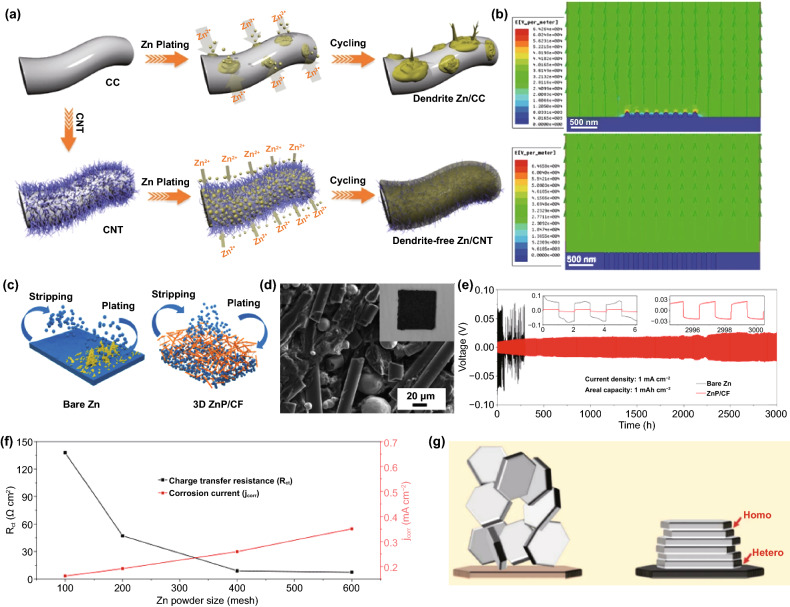
**a** Schematic illustrations of Zn deposition on CC and CNT electrodes; **b** Models of the electric field distributions for a Zn/CC electrode (top) and a Zn/CNT electrode (bottom) after Zn nuclei formation [106]. Copyright 2019, Wiley–VCH. **c** Schematic diagrams of the Zn growth mechanisms on different anode structures; **d** SEM image of ZnP/CF composite electrode. Inset shows the optical pictures of ZnP/CF composite electrode; **e** Voltage profiles of the symmetric cells using Zn foil electrode and 3D ZnP/CF electrode at the current density of 1 mA cm^−2^ and 1 mAh cm^−2^; **f** Plots of the charge transfer resistance and the corrosion current with different particle sizes [107]. Copyright 2020, Elsevier. **g** Scheme illustrating the design principle of epitaxial metal electrodeposition [109]. Copyright 2019, American Association for the Advancement of Science

Besides, the carbonaceous matrix materials have an open 3D skeleton structure; their mechanical flexibility can sufficiently cope with the change in anode volume during cycling, particularly adaptable to shapeable anodes applied to flexible and wearable electronic devices, such as fiber-shaped Zn-ion micro-batteries [102, 103]. Up to now, several excellent research works have developed multiple carbon-based structure anodes, mainly based on highly conductive carbon fiber matrix, including carbon cloth [104], graphite felt [105], carbon nanotubes [106], etc. Impressively, the application of flexible 3D carbon nanotubes (CNT) scaffold on Zn anode has attracted much attention (Fig. 11a) [106]. The interconnected CNT formed on the carbon fiber cloth can successfully lower the energy barrier of Zn nucleation, and the uniform electric field distribution ensures the reversible plating/stripping of the dendrite-free anode (Fig. 11b). Generally, Zn on a carbon-based host is formed in situ in advance by many methods, such as electrodeposition or vapor deposition. Recently, by using a vacuum filtration process, active Zn powder can also be compounded on the carbon network to form a 3D porous Zn anode (Fig. 11c) [107]. It can be understood that point-to-point contact between Zn powder particles may form a vulnerable conductive network. During the Zn stripping process, owing to the short electron pathway, the contact points are likely to be dissolved first, and the Zn powder particles will easily lose contact with the electrode matrix and cause Zn death [108]. However, the carbon fiber skeleton can act as a binder to firmly bond the Zn powder, which can effectively reduce the capacity loss caused by Zn dissolution (Fig. 11d). Although not as large as the specific surface area of nanoscale Zn by in situ nucleation and growth, a large number of micron-scale pores between powder particles or carbon fiber skeletons in the electrode provide enough space for Zn deposition and dendrite growth, resulting in stable anode performance (Fig. 11e). The adjustable surface area of Zn powder has practical significance for commercial production. It is worth beware that the Zn charge transfer resistance decreases with the particle size of the Zn powder, implying that the smaller particle size Zn powder has a higher reactivity. Still, at the same time, it is accompanied by enhanced hydrogen evolution and corrosion reaction (Fig. 11f). Thus, the Zn powder particle size in the carbon fiber framework should be designed reasonably. Additionally, it has been reported that a similar atomic arrangement between graphene and Zn metal results in low lattice mismatch [109]. During the deposition process, Zn^2+^ ions will heteroepitaxially nucleate along the graphene crystal plane and grow in a strain-free state, and then Zn^2+^ ions continue to deposit homogeneously epitaxially on the newly formed Zn metal layer (Fig. 11g). This Zn deposition pattern that locks the crystal orientation relationship can fundamentally eliminate the growth of dendrites. The reversibility of the epitaxial Zn anode guided by the graphene substrate is greatly improved, and the CE still exceeds 99% even after 1000 cycles. Although there is a lack of strong evidence to prove that the Zn deposits on graphene sheets are oriented, this method of manipulating the anode host to control the preferential Zn deposition direction deserves further study [110].

**Fig. 12:**
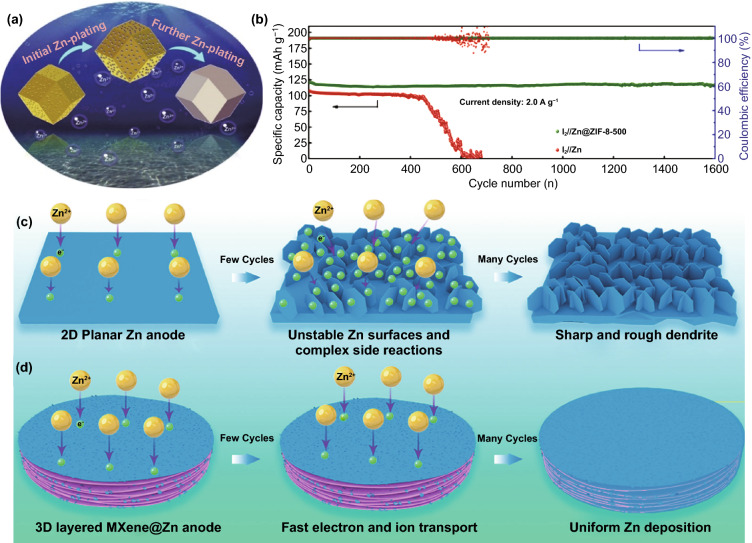
**a** Schematic illustration of the Zn plating on the ZIF-8–500 electrode; **b** Electrochemical performances of the I_2_//Zn@ZIF-8–500 full cell at a current density of 2.0 A g^−1^ [111]. Copyright 2019, Elsevier. Schematic of morphology evolution for **c** bare Zn and **d** Ti_3_C_2_T_x_ MXene@Zn anode during the stripping/plating process [112]. Copyright 2019, American Chemical Society

In addition to metal-based and carbon-based anode hosts, there are other types of anode hosts, such as MOF-derived material [111]: ZIF-8–500, formed by annealing treatment at 500 °C and then undergoing a thermal reduction (Fig. 12a). On the one hand, the trace amount of Zn^0^ in the porous framework can serve as a uniform nucleus for subsequent Zn deposition, thus alleviating the problem of Zn dendrite growth. On the other hand, the high HER overpotential can effectively slow down water decomposition. With the assistance of ZIF-8–500, the CE of the anode was close to 100%, and the Zn iodine rechargeable battery exhibited a long life of 1,600 cycles (Fig. 12b). Moreover, due to the unique layered structure and high conductivity, MXene is a suitable material for the anode host. Ti_3_C_2_T_x_ MXene was proven to have the effect of regulating Zn deposition behavior (Fig. 12c, d) [112]. The increased specific surface area and enhanced hydrophilicity can ensure low current density and high ion mobility, so no Zn dendrites are observed after long charge–discharge cycles. It can be investigated that the layered structure of MXene can introduce a variety of zincophilic and chemically inert seed crystals as Zn nucleation sites to induce more uniform Zn deposition, which has been similarly reported in the modification of Li metal-containing batteries [113, 114]. Recently, it was reported that MXene paper was modified with antimony (Sb) to be used as a Zn anode host. Benefiting from zincophilic Sb seeds and MXene architecture, stable battery performance can be achieved. Similarly, Cu or Ni can replace Sb as a seed crystal to modify MXene paper [115].

### Alloying Anode

Alloy refers to mixing metal with other metals or nonmetals to obtain a material with metallic properties. Zn can be fused with various elements to form Zn-based alloys, classified into binary alloys, ternary alloys, and multi-element alloys according to the number of their components. In addition to Ga–In–Zn [116, 117] and Zn–Sn–Pb [118] alloys, the Zn alloys currently reported on mild aqueous Zn anodes are mainly binary alloys, such as Cu–Zn [89, 119, 120], Ag–Zn [89, 121–123], Zn–Al [124], Zn–Mn [125], Zn–Sb [115], and Zn-P [69]. The interaction between alloy components will form an alloy phase with a specific structure and composition, which can be divided into solid solution and intermetallic compound [126]. Even though the component species are the same, the alloy properties may differ due to different alloy phases formed. This greatly enriches the categories of alloys with other properties. The electrochemical alloying reaction of Zn includes a restructuring reaction and solid solution reaction [127]. Compared with the former, the latter does not require a significant phase change, resulting in less demand for additional activation energy, which means lower charge/discharge voltage hysteresis. Instead of standard Zn stripping/plating on the metal foil surface, the dealloying/alloying reactions involved in the solid solution reaction should be the mechanism that indicates the inward-transfer and reversible extraction of Zn atoms in the Zn alloy [128]. Therefore, based on the stable solution reaction, the Zn alloy anode with different alloy phases can significantly enhance the battery performance.

**Fig. 13:**
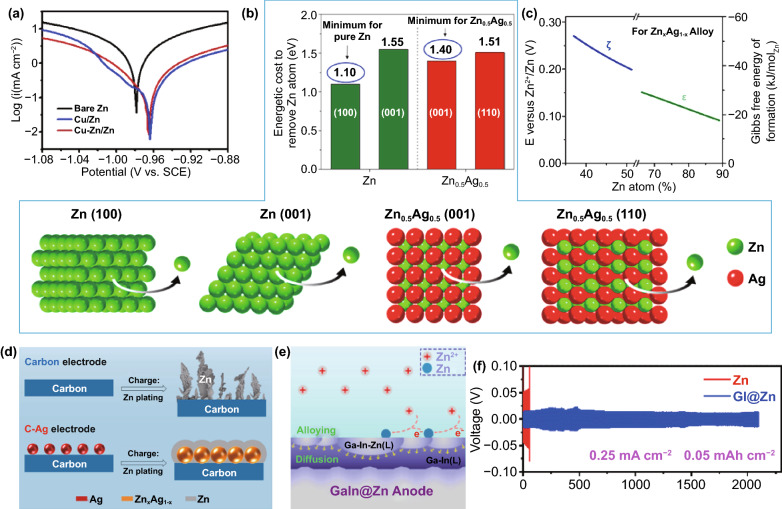
**a** Linear polarization curve of Cu/Zn and Cu–Zn/Zn electrode in 3 M ZnSO_4_ electrolyte [119]. Copyright 2020, Elsevier. **b** DFT simulation results showing the energetic cost of removing a Zn atom from the pure Zn metal and Zn_0.5_Ag_0.5_ alloy. Constructed models: Zn with 001 and 100 surfaces; Zn_0.5_Ag_0.5_ with 110 and 001 surfaces; **c** Calculated Gibbs free energy of formation at room temperature of Zn, *ζ*- and *ε*-Zn_x_Ag_1−x_ alloy phases and the corresponding electrochemical potential shift of Zn^2+^/Zn_x_Ag_1−x_ compared with that of Zn^2+^/Zn. **d** Schematic of Zn deposition on the (top) carbon paper substrate and (bottom) carbon paper slurry coated with Ag nanoparticles [121]. Copyright 2021, American Chemical Society. **e** Dendrite-free GaIn@Zn anode by alloying–diffusion synergistic strategy; **f** Voltage profiles of symmetric cells using bare Zn foil and GaIn@Zn at a current density of 0.25 mA cm^−2^ [116]. Copyright 2021, American Chemical Society

The addition of electrochemically inert metals not only changes the physical properties but also enhances electrochemical performance. Reviewing the Zn alloy anode, Cu and Ag species can improve the corrosion resistance of Zn alloys, and the corrosion potential is close to that of the pure metal introduced (Fig. 13a) [18]. However, this conclusion is affected by many factors, such as element ratio, microstructure, and the alloy phase, making it difficult to explain the detailed corrosion resistance mechanism [18, 24]. A relatively simple strategy has been proposed that the enhanced corrosion resistance of alloys can be analyzed from the perspective of energy supported by DFT (Fig. 13b) [121]. Comparing the minimum energy cost of removing Zn atoms from pure Zn and Zn_0.5_Ag_0.5_, 1.10 eV for pure Zn with 100 crystal plane is lower than 1.40 eV for Zn_0.5_Ag_0.5_ with a 001 crystal plane, implying that the Zn atoms in Zn_0.5_Ag_0.5_ have lower reactivity. The more energy cost for Zn stripping from the Zn_x_Ag_1−x_ alloy corresponds to a higher redox potential (Fig. 13c); thus, the Zn_x_Ag_1−x_ alloy has better corrosion resistance. Similarly, the alloy’s suppression strategy for dendrites can also be analyzed from the nucleation energy. Compared with pure Zn metal, Zn_x_Ag_1−x_ alloys have a lower nucleation energy barrier (Fig. 13c). This indicates that after Zn is reduced, it will spontaneously form an alloy phase with Ag lattice through solid solution reaction, rather than accumulating at sites containing only Zn species (Fig. 13d) [89, 121, 122]. However, excessive Zn deposition will still form a Zn layer on the alloy surface, inducing dendrite growth. Differently, liquid alloys have high fluidity and deformability and can avoid the formation of dendrites through a self-healing mechanism [116, 117]. The high Zn affinity of liquid Ga–In alloy enables the preferential formation of Ga–In–Zn ternary alloy (Fig. 13e). When the Zn species in the alloy reaches saturation, excess Zn spontaneously accumulates under the alloy layer instead of on the surface. Thus, the alloy surface can be maintained smooth, and dendrites cannot be formed on the alloy surface. Collaborating with the high HER overpotential of the alloy and the low impedance of the liquid–liquid interface, the symmetrical battery assembled by the gain anode can be cycled stably for 2000 h with a capacity of 0.05 mAh cm^−2^ at a current density of 0.25 mA cm^−2^ (Fig. 13f).

**Fig. 14:**
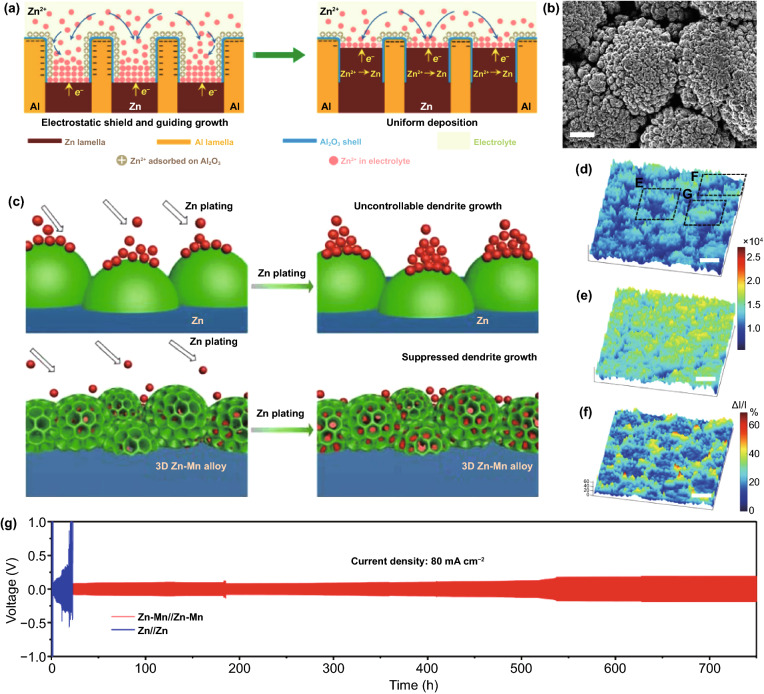
**a** Schematic illustrations of eutectic Al–Zn for uniform Zn deposition [124]. **b** SEM image of Zn–Mn alloy; **c** Schematic illustration of Zn plating processes on Zn anode and Zn–Mn anode; The images of 3D Zn–Mn alloy by in situ optical microscope before **d** and after **e** Zn plating, **f** was calculated by (d–e)/e = (ΔI/I); **g** Long-term galvanostatic cycling performance of symmetric Zn–Mn and pristine Zn cells at a current density of 80 mA cm^−2^ (areal capacity, 16 mAh cm^−2^; electrolyte, 2 M ZnSO_4_ in seawater) [125]

Based on the above analysis, it can be concluded that the alloy mainly suppresses side reactions and dendrite growth by increasing the HER overpotential and reducing the nucleation energy barrier. However, Zn alloys with more active metals than Zn may suffer from the competitive reaction of metal species and corrosion problems, which means that other compensable properties are needed to serve stable Zn anode. It has been proposed that the eutectic Zn_88_Al_12_ alloy with alternating Al and Zn layers can induce uniform Zn deposition by spontaneously constructing an insulating frame, which can be detailed from the two stages of Zn stripping and plating (Fig. 14a) [124]. In the initial stripping stage, since the standard equilibrium potential of Al^3+^/Al (−1.66 V) is lower than that of Zn^2+^/Zn, Al will react in preference to Zn and be converted to Al_2_O_3_. Subsequently, the dense and insulating Al_2_O_3_ passivation layer protects the anode from further oxidation so that the Al–Zn alloy anode can maintain stability. During the plating process, the positive electrostatic shielding layer formed around the plate prevents the Zn nucleation on the surface of Al_2_O_3_, thereby guiding Zn deposition in the correct position.

It is worth noting that the alloy preparation approaches in the laboratory mainly involve electrodeposition and chemical replacement [126], which results in a relatively single appearance of the alloy. Different microstructures of alloys containing the same element species have other effects on anode performance. By developing a reasonable preparation process, the alloy can be designed into a favorable structure to further enhance the performance of the anode. Impressively, a co-electrodeposition process of various ions has been proposed. The evolution of H_2_ bubbles at the solid–liquid interface can lead to the formation of a 3D anode structure [125]. Applying this strategy, the cauliflower-like 3D Zn_3_Mn alloy can be obtained in the solution containing Zn^2+^ and Mn^2+^ ions (Fig. 14b) [125]. The porous morphology with a large specific surface area promotes effective Zn^2+^ ion migration. Additionally, due to the relatively high binding energy on the alloy surface, Zn nucleation and growth are induced and regulated (Fig. 14c). During the Zn deposition process, the deposition rate in the trenches is much larger than that in the protruding area, which minimizes the formation of dendrites (Fig. 14d–f). Benefiting from these advantages, the Zn_3_Mn alloy anode has ultra-high reversibility. Even in the seawater-based electrolyte, despite the interference of various impurity ions, it still shows high stability and reliability (Fig. 14g). Note that this co-electrodeposition strategy can be extended to other alloy systems by adjusting the composition of the deposition solution, the applied deposition current or voltage, and the deposition time. Therefore, it is recommended that while introducing more element species to the alloy anode, the alloy structure can be designed by developing the alloy preparation process.

### Intercalation Anode

**Fig. 15:**
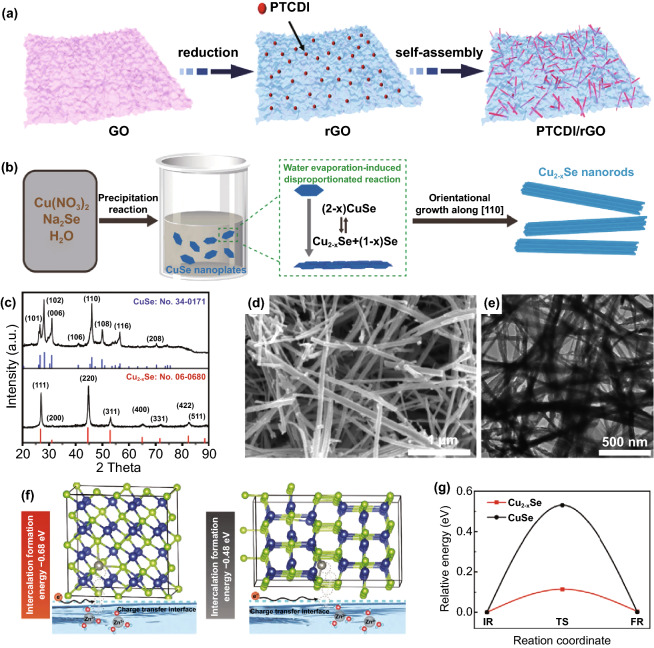
**a** Schematic illustration of preparing the PTCDI/rGO composite [131]. **b** Schematic illustration of the formation of the Cu_2−x_Se nanorods; **c** XRD patterns of Cu_2−x_Se and CuSe; SEM **d** and TEM **e** images of the Cu_2−x_Se nanorods; **f** Charge transfer process at the reaction interface and intercalation formation energy of Cu_2−x_Se (right) and CuSe (left); **g** Diffusion barrier of Zn^2+^ ion in Cu_2−x_Se and CuSe [132]. Copyright 2020, Wiley–VCH

The successful application of intercalated anodes in lithium batteries has attracted more and more attention. Unlike the direct deposition/stripping of charged ions on the conventional metal anode, the lithium ions participate in battery storage and release of energy through intercalation/deintercalation on intercalation anodes [129]. Since there is no metal electrode, dendrite growth and corrosion are eliminated. This unique anode structure and its working mechanism have also inspired people to explore the application of intercalation anodes in mild aqueous ZIBs. However, there are few reports on intercalated Zn anodes, and the properties of the proposed materials are generally unsatisfactory. Due to the lack of high-performance materials and the ambiguity of the Zn storage mechanism, low energy density is one of the biggest challenges of intercalation anodes. The intercalation electrode materials that have been reported are mainly collected in Chevrel phases, transition metal oxides or sulfides, complex transition metal compounds, organic compounds, etc. [130]. There have been some reviews detailing the development of intercalation electrodes in mild aqueous electrolytes. Based on this status quo, recently, a high-rate intercalation anode (PTCDI/rGO) could be obtained by hybridizing perylene-3,4,9,10-tetracarboxylic diimide (PTCDI) and reduced graphene oxide (rGO) (Fig. 15a) [131]. During the charge and discharge, the PTCDI organic electrode provides high electron mobility and prevents the dissolution of discharge products. Besides, the porous structure caused by rGO maintains a wide range of active sites and ion diffusion paths. In addition, Yang et al. [132] proposed mixed-valence Cu_2-x_Se as an intercalation anode to solve the problems of Zn dendrite growth and electrolyte decomposition (Fig. 15b-e). Stable material structure, abundant cation sites, and high conductivity ensure the rapid insertion and extraction of Zn^2+^ ions. The low-valence copper in the electrode material is not only conducive to the generation of suitable intercalation formation energy (Fig. 15f) but also can reduce the Zn^2+^ ion diffusion barrier (Fig. 15g). Low-valence copper can regulate the active sites of Zn^2+^ ion storage and optimize the electronic interaction between active sites and intercalated Zn^2+^ ions. When matched with Zn_x_MnO_2_, the full battery exhibited an extremely long cycle life of over 20,000 cycles at 2 A g^−1^.

### Liquid Electrolyte

As the medium for conducting ions between the anode and cathode, the liquid aqueous electrolyte profoundly affects anode performance in mild aqueous ZIBs. A variety of modified liquid electrolytes have been proposed, which reflect different anode control strategies. This section mainly discusses the regulation strategies of liquid electrolytes on Zn deposition behavior, including weakening of solvation effect, suppression of 2D diffusion, formation of electrostatic shielding layer, and formation of in situ SEI layer.

#### Weakening of Solvation Effect

**Fig. 16:**
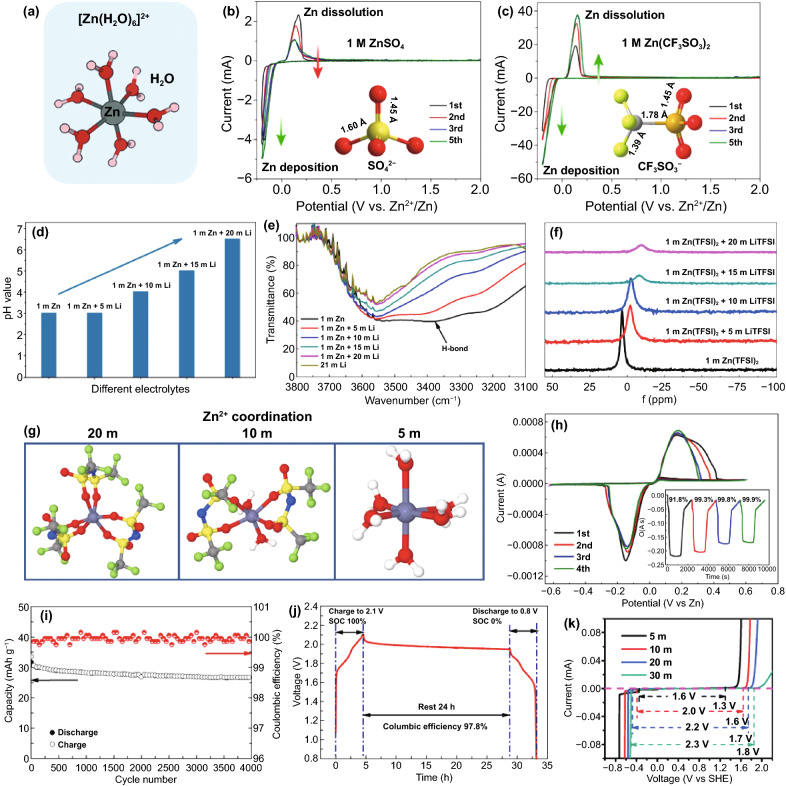
**a** Coordination environment of Zn^2+^ in water [54]. Copyright 2020, Wiley–VCH. Cyclic voltammograms of Zn electrode in aqueous electrolyte of **b** 1 M Zn(CF_3_SO_3_)_2_ and **c** 1 M ZnSO_4_ at the scan rate of 0.5 mV s^−1^ between − 0.2 and 2.0 V [133]. Copyright 2016, American Chemical Society. **d** The pH values of the electrolytes with varying LiTFSI concentrations; **e** The progression of FTIR spectra with salt concentration between 3,800 and 3,100 cm^−1^; **f** The change with salt concentration of chemical shifts for ^17^O nuclei in solvent (water); **g** Representative Zn^2+^ solvation structures in the electrolytes with 1 M Zn(TFSI)_2_ and three concentrations of LiTFSI (5, 10, and 20 M); **h** Cyclic voltammogram of Zn plating/stripping in a three-electrode cell using a Pt disk (2 mm in diameter) as the working and Zn as the reference and counter electrodes at a scan rate of 1 mV s^−1^. Inset: chronocoulometry curves; **i** Cycling stability and CE of the Zn/LiMn_2_O_4_ full cell in HCZE at 4 C rates; **j** Storage performance evaluated by resting for 24 h at 100% state of charge (SOC) after ten cycles at 0.2 C, followed by full discharging [134]. Copyright 2018, Springer Nature. **k** Electrochemical stability window of the ZnCl_2_ electrolyte at different concentrations [135]. Copyright 2018, Royal Society of Chemistry

As mentioned above, in a mild electrolyte, Zn^2+^ ions can cooperate with water molecules to form [Zn(H_2_O)_6_]^2+^ with a sheath structure, the bulky solvation structure is not conducive to the migration and deposition of Zn^2+^ ions, resulting in reduced battery performance (Fig. 16a). It is necessary to reduce the degree of solvation. There are various influencing factors that affect the solvation structure of Zn^2+^ ion in liquid aqueous electrolyte, such as species of anion salt, electrolyte concentration, and additives.

Generally, in Zn salt electrolytes, anions may affect the solvation process of Zn^2+^ ions. The commonly reported anion salt species currently mainly involve SO_4_^2−^, CF_3_SO_3_^−^, TFSI^−^, CH_3_COO^−^, NO_3_^−^, Cl^−^, etc. [52]. Although the most widely used SO_4_^2−^ anion has a stable structure and excellent compatibility with Zn anodes, it cannot effectively alleviate the solvation effect, which has become resistant to further development. It has been reported that in the Zn(CF_3_SO_3_)_2_ electrolyte, the bulky CF_3_SO_3_^−^ anions with a single charge can reduce the number of water molecules in the solvent sheath around the Zn^2+^ ion, and the desolvated Zn^2+^ ion can achieve faster transfer, thereby increasing the Zn^2+^ ion migration and charge transfer rate (Fig. 16b, c) [133]. Therefore, a battery with a reduced solvation effect can be obtained by rationally selecting the anion species of the electrolyte.

Increasing the salt concentration in the electrolyte to reduce the contact chance of Zn^2+^ ions with the surrounding water seems to solve this problem effectively. As a proof of concept, Wang et al. [134] found that as the concentration of LiTFSI increases, the pH of the electrolyte gradually increases and finally stabilizes at about 7, which meant that the interaction between water and Zn^2+^ ions was inhibited (Fig. 16d). According to Fourier transform infrared (FTIR) spectra (Fig. 16e), the 3414 cm^−1^ peak, which reflected the hydrogen bond, disappeared completely at a salt concentration of 10 M, indicating that the hydrogen bond network in the water had been extensively destroyed. This phenomenon had also been confirmed in NMR (nuclear magnetic resonance) spectra (Fig. 16f). Based on molecular dynamics (MD) simulation, it could be recognized that ultra-high-concentration electrolytes containing 1 M Zn(TFSI)_2_ and 20 M LiTFSI completely changed the coordination environment of Zn^2+^ ions, in which Zn^2+^ ions only coordinated with TFSI^−^, while water molecules were surrounded by TFSI^−^ (Fig. 16g). At the same time, the high concentration of electrolytes reduces water activity and water-induced side reactions. Hydrogen evolution and corrosion were dramatically suppressed by eliminating the step of desolvation and blocking the contact of water with the anode interface. The assembled battery not only exhibited high CE in the electrochemical test but also had a high-capacity retention rate during storage (97.8% after 24 h) (Fig. 16h–j). Similarly, Zhang et al. [135] did not observe the formation of anode by-products in the “water-in-salt” electrolyte with 30 M ZnCl_2_, in which the solvated structure will be converted to [ZnCl_4_]^2−^. The electrochemical stability window of the ZnCl_2_ electrolyte was widened along with the decrease in the hydrogen evolution potential due to the increase in concentration, which improved the CE of Zn plating/stripping as well (Fig. 16k). However, in addition to the increasing cost and reducing battery energy density, the electrolyte with an excessively high concentration exposes the characteristics of high viscosity, poor wettability, and low ionic conductivity, which limits the commercial development of Zn^2+^ ion batteries. Nevertheless, this strategy of applying high-concentration electrolytes still has a great practical effect. For example, appropriately increasing the electrolyte concentration is beneficial to the improvement in battery performance. We need to explore the appropriate concentration of electrolytes to achieve the optimization of comprehensive benefits.

**Fig. 17:**
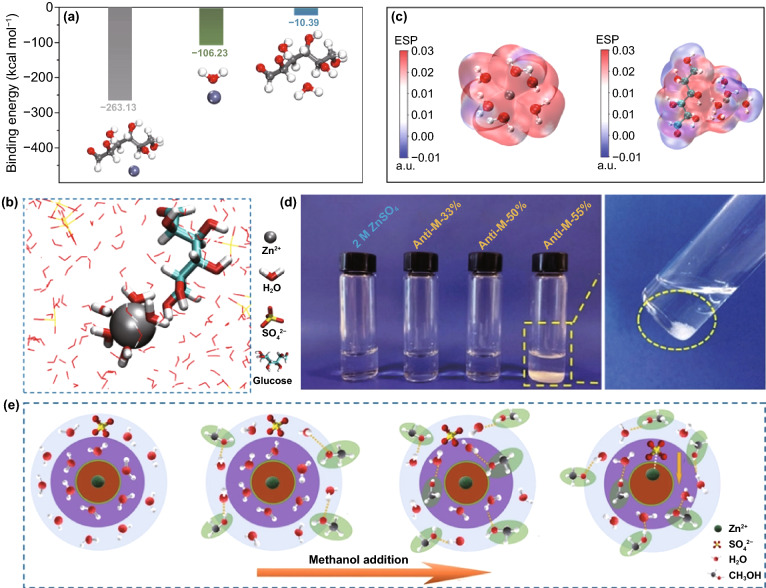
**a** Binding energy for Zn^2+^ with different compounds (glucose and H_2_O) under DFT calculation; **b** Partial enlarged 3D snapshot representing Zn^2+^ solvation structure, obtained from MD simulations of ZnSO_4_–glucose system; **c** Electrostatic potential mapping of the original Zn^2+^–6H_2_O (left) and glucose–Zn^2+^–5H_2_O (right) solvation structures [136]. Copyright 2021, Wiley–VCH. **d** Preparation of methanol-based antisolvent electrolytes; inset shows recrystallization of ZnSO_4_ in antisolvent electrolyte of 55% methanol; **e** Schematic of changes in the Zn^2+^ solvent sheath, together with methanol addition [140]. Copyright 2020, Wiley–VCH

Some additive molecules can interact strongly with Zn^2+^ ions to adjust the Zn^2+^ coordination environment; glucose additive was incorporated into the H_2_SO_4_ electrolyte to form a mixed electrolyte [136]. Experiments and theoretical simulations confirmed that Zn^2+^ ions exhibited a stronger binding interaction with glucose than water molecules (Fig. 17a). Hence, glucose can enter the primary solvation shell of Zn^2+^ ions, replacing part of the water molecules in the solvent sheath around Zn^2+^. Thus, Zn^2+^ mainly existed in the form of glucose–Zn^2+^–5H_2_O solvation structures (Fig. 17b). The significantly decreased electrostatic potential value indicates that the electrostatic repulsion around Zn^2+^ ions can be relieved, which is beneficial to their rapid migration (Fig. 17c). Likewise, some other additives recently reported, such as glycerol [137], acetonitrile (AN) [138], and ethylene glycol (EG) [139], can also reduce the degree of hydration. Slightly different, some additives can directly interact with water molecules. For example, considering that recrystallization or delamination occurs in the ZnSO_4_ electrolyte with some liquid alcohol, Hao et al. [140] introduced an antisolvent strategy to the electrolyte. Due to the small molecular volume and high dielectric constant, methanol is added to the ZnSO_4_ electrolyte as an antisolvent (Fig. 17d). The methanol molecules initially attract free water molecules from the solvation of Zn^2+^ through hydrogen bonds (Fig. 17e). As the methanol concentration increases, methanol molecules will be inserted into the outer and inner layers of the Zn^2+^ solvation sheath. The exposed Zn^2+^ ions will recombine with SO_4_^2−^ ions, which means that methanol can reduce water activity and disturb the coordination balance between water and Zn^2+^ ions.

#### Suppression of 2D Diffusion

**Fig. 18:**
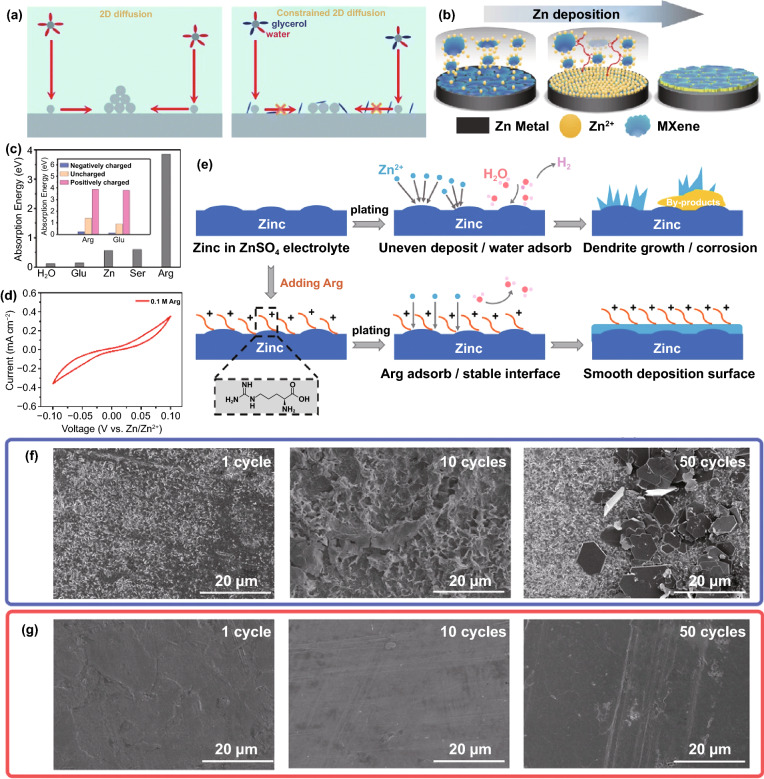
**a** Schematics of the Zn^2+^ diffusion and reduction processes on the bare Zn electrode in aqueous and hybrid electrolytes, showing that the surface diffusion is constrained in the hybrid electrolyte [137]. Copyright 2021, Royal Society of Chemistry. **b** Schematic illustration of the effect of MXene additive on the Zn deposition process [144]. **c** Absorption energy of H_2_O, Glu, Zn, Ser, and Arg on Zn surface in mildly acidic electrolyte, respectively. Inset is absorption energy of Arg and Glu with positively charged, uncharged, and negatively charged on Zn surface, respectively; **d** CV curve of Zn symmetric cell with 0.1 M Arg solution at 5 mV s^−1^. **e** Schematic illustration of Zn plating behavior with and without Arg additive; surface morphology of Zn electrode at 1st, 10th, and 50th cycle **f** in bare ZnSO_4_ electrolyte and **g** in ZnSO_4_ + Arg electrolyte [145]. Copyright 2021, Wiley–VCH

Additives involving some organic molecules can be adsorbed on the Zn anode surface, thereby inhibiting the 2D diffusion of Zn^2+^ ions. Adsorption interactions may be derived from electrostatic induction and chemical bonds between additives and Zn metal. The strong adsorption interaction is favorable to a stable anode interface. It has been reported that glycerol [137], polyethylene glycol (PEG200) [141], polyethylene oxide (PEO) [142, 143], and other organic molecular additives can achieve adsorption on the anode surface. These additives adsorbed on the metal surface are similar to the artificial non-conductive modified layer. On the one hand, they act as a physical barrier to prevent the surface migration of Zn^2+^ ions. Therefore, Zn^2+^ ions form a large number of tiny nuclei at the initial contact site with the metal (Fig. 18a). On the other hand, the groups of some additives can adsorb Zn^2+^ ions to inhibit surface diffusion, and Zn preferentially nucleates around the additives fixed on the anode surface (Fig. 18b) [142–144]. Resulting from the suppressed 2D diffusion, the subsequent Zn deposition will grow a dense and smooth Zn layer. In a recent report of amino acids as additives [145], positively charged amino acids, especially arginine (Arg), will preferentially undergo anodic interface adsorption due to their higher adsorption energy than H_2_O and Zn (Fig. 18c). The adaptive absorption layer of Arg, which belongs to the capacitive adsorption behavior (Fig. 18d), can adapt to the dynamic interface changes and effectively adjust the interface charge during the Zn electroplating/stripping process. According to the chronoamperometry curve, the suppressed 2D diffusion leads to a uniform Zn^2+^ ion flux so that the Zn^2+^ ions in contact with the anode surface tend to react directly to alleviate continuous dendritic growth (Fig. 18e–g).

#### Formation of Electrostatic Shielding Layer

**Fig. 19:**
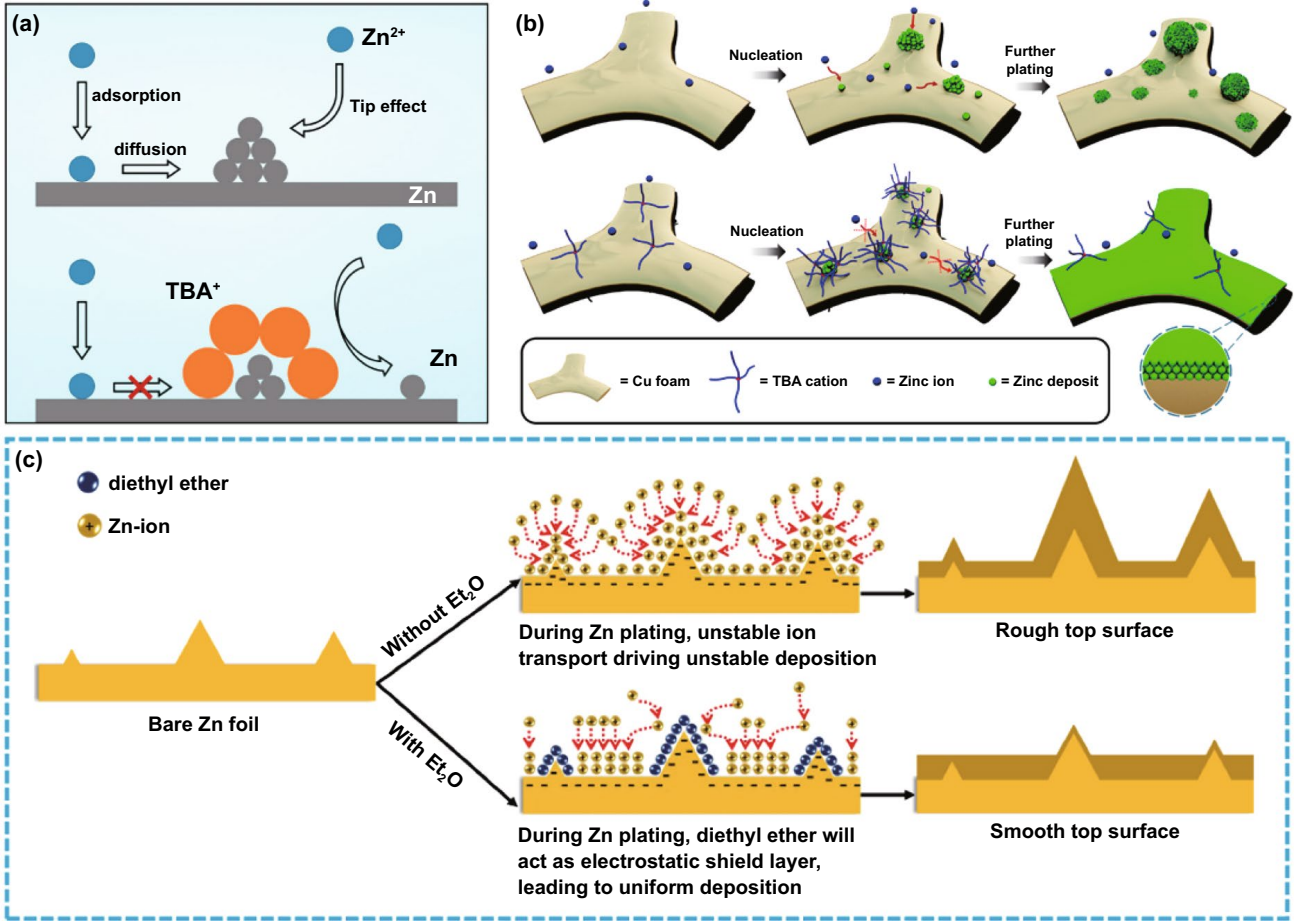
**a** Schematics of the Zn^2+^ ion diffusion and reduction processes on electrodes in 2 M ZnSO_4_ electrolyte (top) without and (bottom) with 0.05 mM TBA_2_SO_4_; **b** Schematic illustrations of Zn deposition on Cu foam without or with TBA_2_SO_4_ as an electrolyte additive [148]. Copyright 2020, American Chemical Society. **c** Schematics of morphology evolution for Zn anodes in mild aqueous electrolyte with and without Et_2_O additive during Zn stripping/plating cycling [150]. Copyright 2019, Elsevier

A part of additives can form an electrostatic shielding layer at the tip to inhibit the growth of dendrites (Fig. 19a). During the deposition process, additional cations compete with Zn^2+^ ions for adsorption, and the one with a lower reduction potential in the additives will be preferentially adsorbed on the initial tip, forming electrostatic shielding, thereby changing the subsequent Zn deposition behavior [146, 147]. As a proof of concept, tetrabutylammonium sulfate (TBA_2_SO_4_) has been used as an electrolyte additive to regulate Zn deposition (Fig. 19b) [148]. During the plating process, the reduction potential of TBA^+^ cations is lower than that of Zn^2+^ ions, so the TBA^+^ cations will preferentially adsorb at the tip, which counteracts the tip effect and further drives Zn^2+^ ions to nucleate around the non-protrusions, thus regulating the deposition behavior of Zn^2+^ ions. Finally, a smooth deposited layer is formed. In addition to TBA_2_SO_4_, some other metal cation-containing additives also have similar effects, such as Na_2_SO_4_ [149]. Moreover, some stable and polar organic molecules, such as ether [150], can also form an electrostatic shielding layer at the tip (Fig. 19c). In the high electric field around the tip, the polarized molecules are easily attracted to the tip. Good chemical stability ensures that organic molecules will not participate in the transfer of electrons. As a result, the Zn deposition behavior can also be controlled.

#### Formation of In Situ SEI

**Fig. 20:**
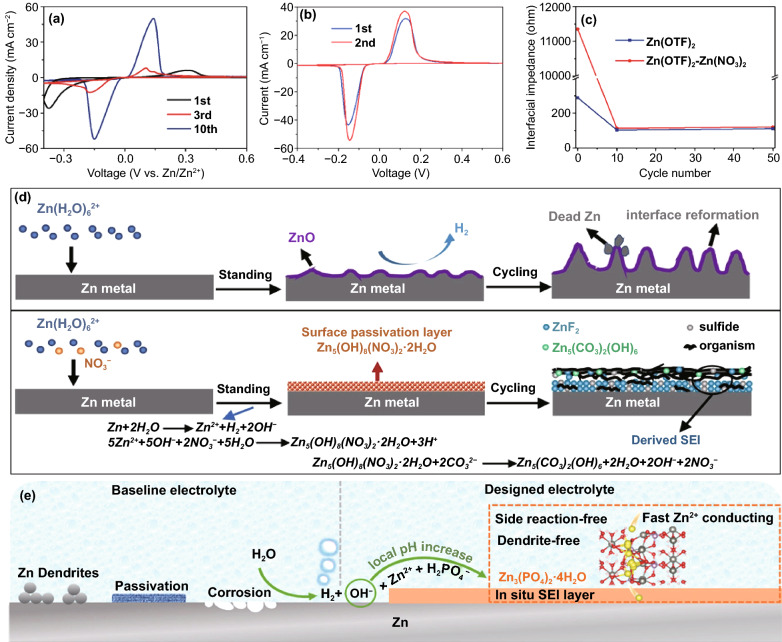
CV curves of Zn‖Ti half cells **a** with and **b** without Zn(NO_3_)_2_ additive; **c** Interfacial impedance measured from Zn‖Zn cells in Zn(OTF)_2_ electrolytes with and without Zn(NO_3_)_2_ additive under cycling; **d** Illustration of surface evolution mechanism in Zn(OTF)_2_ electrolytes with and without Zn(NO_3_)_2_ additive. [151]. Copyright 2021, Wiley–VCH. **e** Schematic illustration of the Zn surface evolution and characterization of Zn electrodes in the baseline and designed electrolytes [155]. Copyright 2021, Wiley–VCH

It has been proved that by adding some additives to the applied electrolyte, a dense and uniform SEI layer can be formed in situ on the anode surface to suppress dendrites and side reactions. As we all know, different from the artificial SEI layer, the in situ SEI layer on the anode surface is generally formed by the decomposition of electrolyte components and/or salt anions when electrolyte is in contact with the anode and during the charge/discharge process. Hence, the in situ formation of SEI is related to complex chemical or electrochemical reaction processes. For example, as an additive, a trace amount of Zn(NO_3_)_2_ is added into the Zn(OTF)_2_ electrolyte [151]. Before Zn plating/stripping process, the local pH increases on the Zn surface due to the H_2_O reduction in HER. And Zn reacts easily with NO_3_^−^/OH^−^ to form Zn_5_(OH)_8_(NO_3_)_2_·2H_2_O passivation layer. During the initial Zn plating/stripping cycles, the typical Zn plating/stripping peaks can be gradually observed in the CV curve of the half-cell (Fig. 20a, b), and the interface impedance approaches that of the additive-free system (Fig. 20c), indicating that there is an activation process. This can be explained by the fact that the passivation layer is converted into Zn_5_(CO_3_)_2_(OH)_6_ layer through metathesis reaction. Then, this layer and salt anions will participate in subsequent reactions. As a result, an electronically insulating and ion-conducting inorganic ZnF_2_-Zn_5_(CO_3_)_2_(OH)_6_–organic bilayer SEI is formed on the anode surface (Fig. 20d). The highly flexible organic outer layer can prevent SEI from cracking due to volume changes. The hydrophobic ZnF_2_ in the inner layer can further enhance desolvation and inhibit H_2_O decomposition and Zn dendrite growth by preventing direct contact between Zn and H_2_O, but allow Zn^2+^ ions to transport. Similarly, ZnF_2_-rich SEI in situ has also been reported in several other works: Introducing trimethylethylammonium trifluoromethanesulfonate (Me_3_EtNOTF) additive into Zn(OTF)_2_ electrolyte can form an SEI composed of ZnF_2_, ZnCO_3_, ZnSO_3_, and polyanions; introducing KPF_6_ additive into the ZnSO_4_ electrolyte can form an SEI composed of Zn_3_(PO_4_)_2_ and ZnF_2_ (ZCS); and introducing ZnF_2_ additive into ZnSO_4_ electrolyte can form F-rich SEI. ZnF_2_ boasting unique merits can increase Zn^2+^ ion migration and deposition kinetics. At the same time, the high interface energy of SEI component with the Zn substrate (such as Zn_3_(PO_4_)_2_/Zn, ZnF_2_/Zn) can suppress dendrite growth by promoting lateral rather than vertical Zn^2+^ migration and deposition [152–154]. Additionally, it can be seen that the change of electrolyte pH generally accompanies the SEI formation process involving H_2_O decomposition. In addition to the above mentioned, the introduction of Zn(H_2_PO_4_)_2_ salt into the Zn(CF_3_SO_3_)_2_ electrolyte can form a hopeite (Zn_3_(PO_4_)_2_·4H_2_O) layer in situ (Fig. 20e) [155]. The formation of this dense SEI is entirely dependent on HER, which can change the local pH. The increased local concentration of OH^−^ anions creates conditions for the spontaneous formation of SEI in situ. This process is to transform the shortcomings caused by HER into advantages. Both this SEI and its formation process can suppress side reactions. In addition, it has recently been reported that in the ZnSO_4_ electrolyte, the introduction of LiCl additives will preferentially form a Li_2_O/Li_2_CO_3_ layer [44]; introducing SnCl_2_ additive into ZnCl_2_ electrolyte can form Sn/Zn_5_(OH)_8_Cl_2_·H_2_O double layer in situ [156]. Based on the above analysis, it can be concluded that in situ SEI is mainly through chemical reaction and mechanical inhibition to alleviate dendrite growth and side reactions. Accordingly, electronic insulation, ion conduction, high Zn/SEI interface energy, and large bulk modulus are essential for a stable in situ SEI. Importantly, the formation process of SEI is similar to the negative feedback effect. The continuous decomposition of electrolyte components and/or salt anions can promote the gradual completion of SEI, which in turn inhibits this process, thereby preventing unrestricted SEI growth. As long as the conditions are sufficient, this process will proceed spontaneously. Almost all reported in situ SEIs have a self-repair function, which is not available in artificial SEIs. Therefore, the strategy of constructing in situ SEI is worthy of in-depth study to realize stable and reversible anodes.

### Non-Liquid Electrolyte

Different from liquid electrolytes, non-liquid electrolytes with unique properties have been designed. Generally, with a decrease in free water content, non-liquid electrolytes have lower ionic conductivity. Fortunately, the controllability of non-liquid components can make up for the shortcomings and achieve performance close to or even better than that of liquid electrolytes. Therefore, a stable and reversible anode interface can be realized, and the high designability of non-liquid electrolytes significantly broadens the working environment range of aqueous ZIBs. This section mainly analyzes solid-state electrolytes, hydrogel electrolytes, and other non-liquid electrolytes.

#### Solid-state Electrolytes

**Fig. 21:**
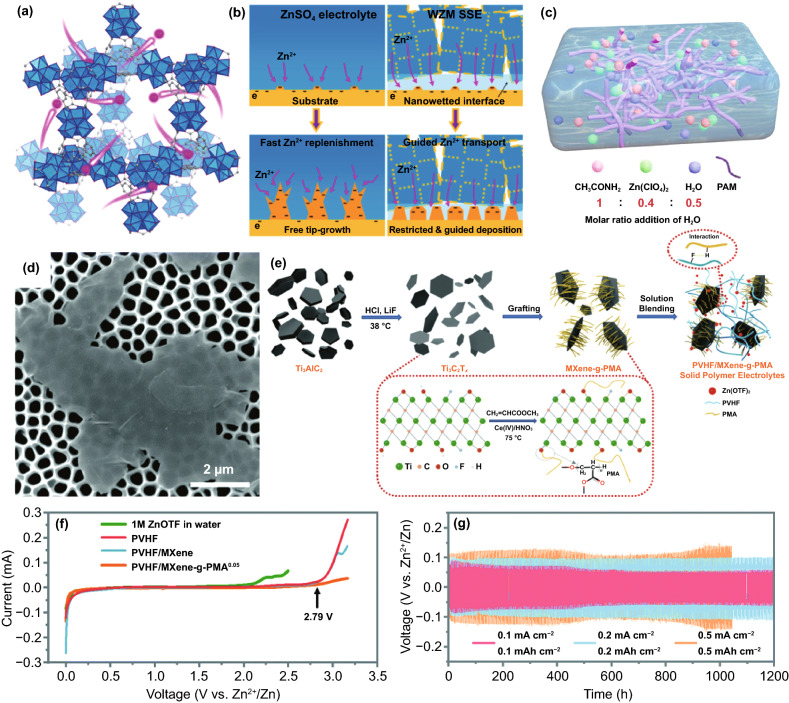
**a** Crystal structure of ZnMOF-808. Blue polyhedrons represent Zr–O clusters, and Zn^2+^ ions are highlighted by pink balls; **b** Proposed mechanism for the different deposition behaviors of ZnSO_4_ aqueous electrolyte (left) and WZM SSE (right) [159]. Copyright 2018, Elsevier. **c** Schematic diagram of IL-PAM composition and structure [161]. Copyright 2021, American Chemical Society. **d** SEM image of MXene-g-PMA; **e** Schematic illustration of the overall preparation process of the SPEs; **f** LSV of the SPEs (scan rate 0.5 mV s^−1^); **g** Galvanostatic Zn plating/stripping in the Zn/Zn symmetrical cells based on different current densities with different plating capacities [162]. Copyright 2021, Royal Society of Chemistry

Due to excellent chemical stability, solid-state electrolytes (SSEs) exhibit high safety. The ion guidance derived from the mechanical structure and surface chemistry of the electrolyte can restrain the Zn dendrites to a certain extent, and the elimination of water can effectively solve the anode interface issues caused by side reactions. However, it is also obvious that the low ion migration rate and high boundary impedance are tricky challenges for all-solid-state electrolytes. Therefore, there are few real all-solid-state electrolytes [20], and most reported solid electrolytes involve a certain amount of liquid plasticizer, such as poly(vinylidene fluoride-hexafluoropropylene)/poly(ethylene oxide) film filled with ionic-liquid-based Zn salt and water-soaked Zn perfluorosulfonate membrane (ZPSAM) [157, 158]. Impressively, the single ion Zn^2+^ SSEs based on the post-synthesis modified MOF-808 have a fixed anion microporous host (Fig. 21a) [159]. At higher humidity, these microporous hosts will absorb water and solvate the Zn^2+^ ions in the pores. Conductive and high-concentration Zn(H_2_O)_6_^2+^ can accelerate ion transfer. Since the water released by desolvation wets the reaction interface and the sub-nano-tunnel guides the deposition site, the Zn deposition morphology is more uniform, dense, and smooth (Fig. 21b).

Due to combining the advantages of the high ionic conductivity of the inorganic electrolyte and the high interface compatibility between the polymer electrolyte and the metal anode, solid polymer electrolytes (SPEs) have attracted significant attention, and they are the most reported SSEs in ZIBs [52, 160]. Recently, based on acetamide/zinc perchlorate hexahydrate ionic liquid, polyacrylamide polymer electrolytes (IL-PAM) have been synthesized (Fig. 21c) [161]. Similar to the electronic insulation artificial modification layer, cross-linked PAM as a 3D skeleton can suppress dendrites. The wettability of water increases the ionic conductivity of IL-PAM. Since water is a trace amount, there is almost no free water in the electrolyte, corresponding to inhibited HER.

Compositing fillers into the polymer matrix can enhance the performance of SPEs. In particular, fillers with rich surface chemistry and a large surface area can significantly increase the ionic conductivity of SPEs. This is mainly achieved by promoting the dissociation of Zn salts, forming ion migration pathways on the filler surface, and acting as plasticizers to reduce polymerization crystallinity and enhance chain migration [162]. Based on this strategy, SPEs containing suitable fillers can show excellent performance without a liquid phase. Thus, the all-solid-state electrolyte has been proposed. For example, the 2D material MXene was chemically grafted with poly(methyl acrylate) to form a composite material (denoted as MXene-g-PMA) (Fig. 21d). As a filler, it was dispersed into poly(vinylidene fluoride-co-hexafluoropropylene) (PVHF) (Fig. 21e). Compared with PVHF and PVHF/MXene, PVHF/MXene-g-PMA had higher ion conductivity. Compared with common aqueous electrolytes, these SPEs can obtain an electrochemical stability window as high as 2.79 V (relative to Zn^2+^/Zn) (Fig. 21f). The SPE-based symmetric battery cycled stably for more than 1000 h without the formation of dendrites at room temperature (Fig. 21g) [162].

#### Hydrogel Electrolyte

**Fig. 22:**
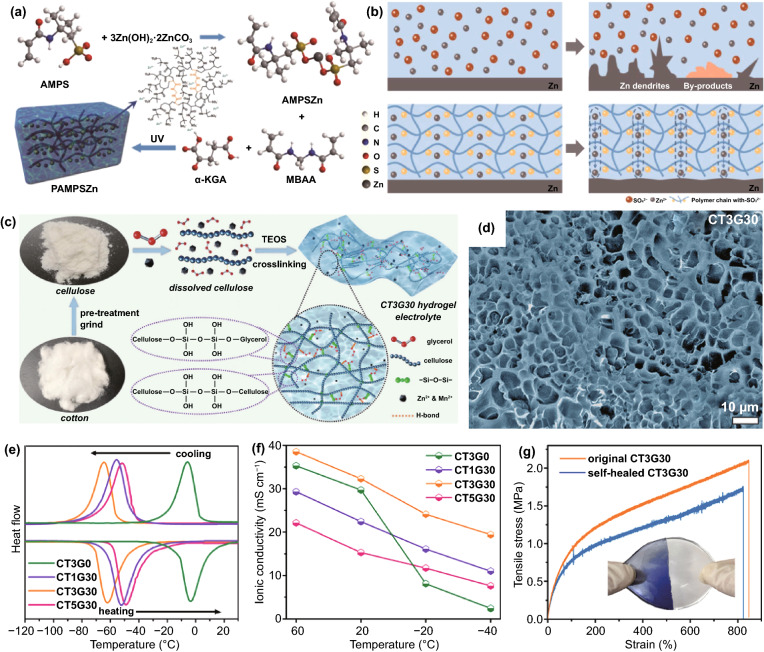
**a** Schematic synthesis of PAMPSZn hydrogel electrolyte; **b** The mechanism of Zn deposition/stripping with ZnSO_4_ aqueous electrolyte and PAMPSZn hydrogel electrolyte [165]. Copyright 2020, Elsevier. **c** Synthesis schematic of the CT3G30 hydrogel electrolyte. **d** SEM image of freeze-dried CT3G30; **e** DSC curves and **f** ionic conductivity values of CT3G30; **g** Tensile σ–ε curves of the original CT3G30 and self-healed CT3G30 [169]. Copyright 2020, Wiley–VCH

With the application of flexible wearable devices, batteries with hydrogel electrolytes have attracted much attention due to their high electrochemical performance and good mechanical properties. The hydrogel network composed of abundant polymer chains through physical/chemical cross-linking macroscopically displays the shape and volume of the quasi-solid [163]. However, since the hydrophilic group of the polymer chain can absorb a large amount of water, the hydrogel electrolyte exhibits a conductivity close to that of a liquid. Meanwhile, water molecules are restricted instead of freely spreading, indicating that HER can be alleviated to a certain extent [164]. The fixed polymer chains play a vital role in ion transfer and Zn deposition. For example, polyanionic hydrogel electrolyte poly-2-acrylamide-2-methyl-1-propanesulfonate zinc (PAMPSZn) was synthesized through ion exchange and free radical polymerization (Fig. 22a) [165]. The fixed polyanionic chain containing –SO_3_^2−^ anions guided and accelerated ion transfer to reduce concentration polarization; PAMPSZn hydrogel electrolyte had high ionic conductivity of 15.6 mS cm^−1^. And the limited contact between -SO_3_^2−^ and Zn metal surface suppressed side reactions. Moreover, the network structure restricted by polyanionic chains served as Zn^2+^ ion transfer channels to facilitate uniform Zn deposition (Fig. 22b). Compared with the ZnSO_4_ aqueous electrolyte, the Zn anode in the PAMPSZn gel electrolyte exhibited a more uniform surface morphology during the plating/stripping process. In addition, Zn alginate (ALG-Zn) [166], gelatin-based SSE (GSE) [167], borax cross-linked polyvinyl alcohol/glycerin (PVA-B-G) [168], etc., have similar functions as hydrogel electrolytes.

Flexible energy storage devices usually face many practical challenges such as extremely low temperature, bending, compression, twisted, or even puncturing. Extraordinarily, superior to liquid or solid electrolytes, hydrogel electrolytes exhibit many practical advantages to solve these problems. All-round hydrogel electrolyte (denoted as CT3G30) has been reported, which employs cotton as the raw material of polymetric framework and tetraethyl orthosilicate (TEOS) as the cross-linker (Fig. 22c, d) [169]. A large number of metal salts (ZnSO_4_ and MnSO_4_) in CT3G30 can lower the freezing point of the aqueous system. Meanwhile, the strong interaction between the hydroxyl groups of the cellulose framework and water molecules can suppress ice crystallization of water molecules through hydrogen bonds. Based on these synergies, CT3G30 exhibits a strong antifreezing ability, implying that this hydrogel electrolyte can be adapted to the operating environment at extremely low temperatures (Fig. 22e, f). Furthermore, CT3G30 possesses very high tensile strength, which derives from proper cross-linking inside the hydrogel. Benefit from a large number of hydrogen bonds that are easy to break and reform as well as Si–O–Si bonds with excellent chain mobility and high reversibility, CT3G30 shows good self-healing ability (Fig. 22g). Also, the high abundance of hydrogen bonds ensures strong adhesion properties. Based on the above characteristics, the practical applicability of CT3G30 hydrogel electrolyte will be greatly enhanced. Similarly, there are many reports of hydrogel electrolytes with unique properties, such as PVA/Zn(CH_3_COO)_2_/Mn(CH_3_COO)_2_(PVA-Zn/Mn) [170] and Zn(CF_3_SO_3_)_2_ polyacrylamide (PAM) [171].

#### Other Non-liquid Electrolyte

**Fig. 23:**
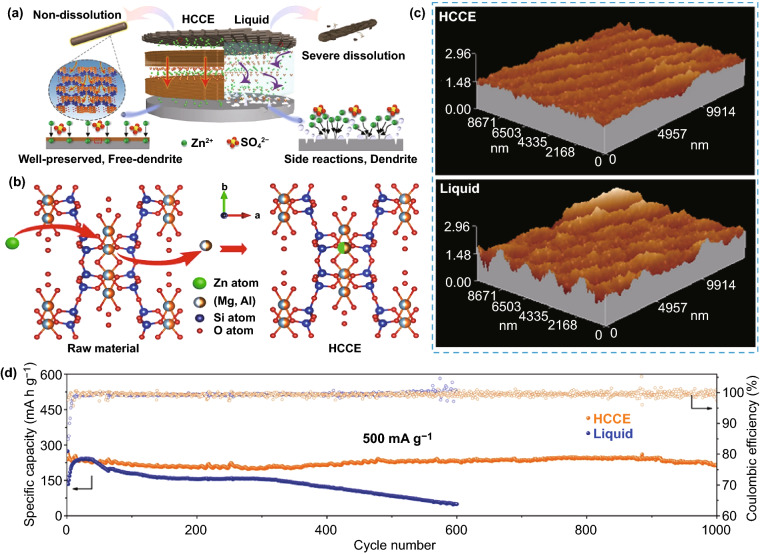
**a** Schematic diagram of interface protection effect in HCCE and liquid electrolyte; **b** Ion exchange diagrams in the HCCE; **c** AFM images of (top) anode of the battery with HCCE and (bottom) the battery with liquid electrolyte cycled for 200 cycles 1000 mA g^−1^; **d** Long-life cycling performance of the cell with HCCE and liquid electrolyte at 500 mA g^−1^ [172]

In addition to hydrogels and solid electrolytes, other materials have also been explored for non-liquid electrolytes. A novel inorganic highly concentrated colloidal electrolyte (HCCE) has been proposed, which is induced by palygorskite nano-inorganic materials [172] (Fig. 23a). The Zn^2+^ ion in the solvent can strongly interact with palygorskite and insert into its inner crystal through an isomorphic substitution reaction. Since this is a reversible process, the metal cations (Zn^2+^ and Mg^2+^) in the solvent and palygorskite will continuously and spontaneously exchange, thereby achieving a dynamic balance of the Zn^2+^ ion concentration. Driven by the concentration gradient in the electrolyte, Zn^2+^ ions eventually migrate between the anode and the cathode (Fig. 23b). On the one hand, the hydroxyl group in palygorskite fixes water molecules by forming hydrogen bonds; on the other hand, the ion exchange process destroys the water solvation sheath of metal cations, both of which are beneficial to reduce the desolvation energy barrier and inhibit side reactions at the anode interface. In addition, by forming an ion-conducting protective layer on the anode surface, HCCE can further suppress dendrites and side reactions (Fig. 23c). As a result, when the anode was Zn and the cathode was MnO_2_, the battery containing HCCE maintained almost 100% capacity after 1000 cycles at 500 mA g^−1^ (Fig. 23d).

### Separator Design

As a crucial part of the battery configuration, the separator regulates the migration of ions in the electrolyte and isolates the two electrodes to prevent direct contact. Thus, the separator also has a significant impact on the performance of the anode. At the same time, considering that dendrite growth may pierce the separator and cause short circuits accordingly, it is also an effective strategy to design a reliable multifunctional separator. At present, although glass fiber (GF) separators are extensively used in aqueous ZIBs in the laboratory, due to the high cost, low ionic conductivity, and terrible mechanical strength, they are still difficult to meet the upgrade requirements of battery performance. Therefore, there is an increasing desire for a novel multi-function separator. Since it is related to all parts of the battery rather than a single mental anode, the research on the separator is of great significance, and it may be the next research hot spot for aqueous batteries.

**Fig. 24:**
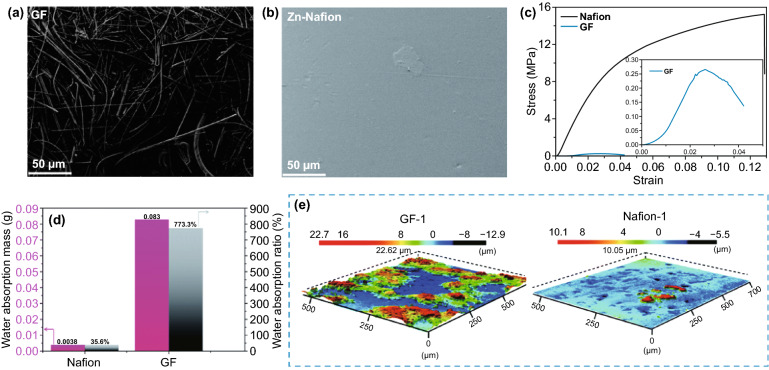
SEM images of **a** GF and **b** Zn-Nafion separators; **c** The stress–strain curves (the inset is a magnified stress–strain curve of the GF separator); **d** The water uptake; **e** The 3D height images for the surface of the (right) GF separator and (left) Zn-Nafion separator after the first cycle [173]. Copyright 2021, Royal Society of Chemistry

Obviously, like the GF separator (Fig. 24a), the traditional porous separators mainly mechanically adjust the mass flux in the solution, which mainly depends on their physical properties, such as mechanical properties, structural morphology, and wettability of the electrolyte. Excellent physical properties of separators play an important role in increasing ion conductivity, homogenizing concentration field, and electric field. Continuing this modification strategy, a Zn^2+^-substituted Nafion separator (Zn-Nafion) was designed (Fig. 24b) [173]. Compared with the GF separator, the Zn-Nafion separator has dominant physical properties: High mechanical strength enables better tolerance to dendrites (Fig. 24c), and low water absorption can reduce electrolyte consumption corresponding to less dissolved oxygen (Fig. 24d). At the same time, the thin structure is beneficial to accelerate the transfer of Zn^2+^ and increase the energy density of the battery. Based on these merits, Zn-Nafion separator promoted the uniform Zn deposition and reduced the concentration polarization (Fig. 24e). Note that the cost of the Zn-Nafion separator is 47.3% lower than that of the glass fiber separator, which means that the commercialization of the former is more promising.

**Fig. 25:**
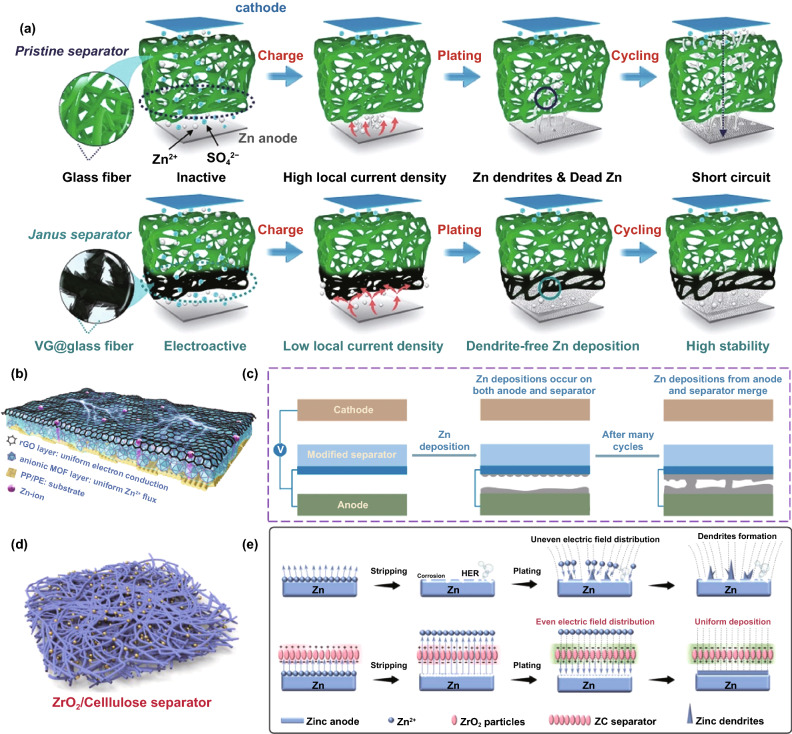
**a** Schematics illustrating (top) a pristine glass fiber separator and (bottom) a Janus separator targeting stabilized Zn anode [174]. Copyright 2020, Wiley–VCH. **b** Schematic illustration for the Janus separator [175]. **c** Schematic illustration of Zn deposition in the contact region of the modified separator and the anode [176]. **d** Schematic illustration for the ZC separator; **e** Schematic illustration of possible migration process of Zn^2+^ when passing through the cellulose and ZC separators [177]. Copyright 2021, Elsevier

In addition to mechanically regulating the Zn^2+^ ion transfer, designing a separator that can interact with ions can further enhance the anode performance. This can be achieved by attaching other attributes to the separator, such as zincophilicity, electronic conductivity, directional polarization electric field, and low lattice mismatch. Correspondingly, a stable Janus separator facing Zn anode is reported (Fig. 25a) [174]. Vertical graphene (VG) carpet was directly grown in situ on one side of the commercial glass fiber separator through plasma-enhanced chemical vapor deposition (PECVD) technology. The 3D VG conductive network with a high surface area can uniform electric field distribution and reduce local current density. By introducing oxygen and nitrogen dopants to improve Zn affinity, the Janus separator can effectively and selectively regulate the Zn^2+^ ions flux. Similarly, compounding MOF/rGO on polypropylene/polyethylene (PP/PE) substrate can obtain a dual-function separator (Fig. 25b) [175]. On the one hand, MOF regulates the uniform Zn^2+^ ion flux with the assistance of its sub-nanometer anion channel. On the other hand, rGO acts as a conductive layer to reduce the potential microscopic difference on the Zn surface, which can enhance the corrosion resistance of the anode. Slightly different, compared to the conductive network, a zincophilic metal layer constructed on the separator facing the anode side, such as metal Sn-modified layer [176], can change the location of Zn deposition (Fig. 25c). Since the anode and the separator modification layer are both electronically conductive and connected, an equipotential surface can be formed between them, implying a uniform electric field between the anode and the separator. The location of Zn deposition is no longer limited to the anode surface. Instead, there is also Zn deposition on the separator surface. The restricted Zn growth direction can eliminate the possibility of dendrites piercing the separator. It can be known that the electronically conductive separator facing the anode side can be regarded as a 3D continuation of the Zn anode, allowing the space to expand to guide Zn deposition. In addition, with the help of the Maxwell–Wagner polarization effect of ZrO_2_, the composite separator made of cellulose nanofibers and ceramic particles can generate a directional polarization electric field, which can guide uniform Zn deposition, increase ion conductivity, and inhibit anion transfer (Fig. 25d, e), thereby stabilizing the Zn anode and suppressing side reactions [177]. As mentioned earlier, low lattice matching can induce the crystal orientation of Zn deposition. Due to the perfect lattice matching between (002)_Zn_ and (002)_GO_, the cellulose/GO composite separator (CG) can also promote the preferential orientation of Zn crystals along the horizontal direction, resulting in non-dendritic Zn electrodeposition [178].

### Other Strategies

Apart from the representative strategies introduced above, some other meaningful attempts have also been continuously made to solve the issue at the anode interface, such as regulating charge/discharge protocols, changing the battery configuration.

**Fig. 26:**
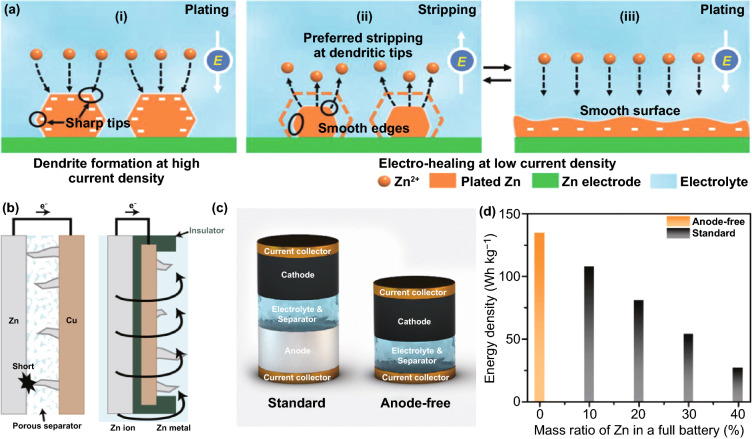
**a** Schematic illustration of the electrohealing process, where the sharp tips of dendrites are passivated into smooth edges and finally produce a smooth electrode surface [29]. Copyright 2019, Wiley–VCH. **b** Schematic representation of (right) conventional frontside- and (left) backside-plating configuration cells [179]. **c** Schematic diagram of standard battery and anode-free battery; **d** Comparison of the energy density of the anode-free and standard batteries [180]. Copyright 2021, American Chemical Society

The various strategies currently proposed to inhibit the growth of dendrites are based on prevention, but these strategies will fail once Zn dendrites are formed. Therefore, a strategy to actively eliminate already formed Zn dendrites in situ is demanded. Yang et al. [29] proposed that the large current density accelerated dendrite formation, while low current density had little effect on dendrite growth. Based on this finding, they offered an electro-healing strategy from the perspective of regulating the charge/discharge protocols. Specifically, they manipulated the anode current density to mediate Zn deposition behavior. The morphology of the anode surface gradually became smooth with the passivation of dendrites during the constant current discharge/charge at a low current density (≤ 1 mA cm^−2^) (Fig. 26a). Obviously, by actively eliminating dendrites, this self-healing strategy can largely extend battery lifespan.

There have been reports of changing the configuration of the battery, such as changing the orientation of the anode reaction interface which can change the direction of dendrite growth to prevent battery short circuit (Fig. 26b) [179]. Currently reported ZIBs usually contain thick anodes caused by excess Zn metal, which results in low energy density. Therefore, the “anode-free” configuration applied in lithium and sodium metal batteries was introduced into ZIBs and further optimized (Fig. 26c). It is coating the Cu current collector with a modified layer of carbon nano-disks without carbon nanoparticles as Zn nucleation sites. In the initial cycle, since there is no Zn metal on the anode side, it is necessary to charge the anode first. The Zn involved in the cycle on the anode comes entirely from the electrolyte and cathode, which means that the Zn on the anode is zero excess. Thus, ZIBs with high energy density can be obtained (Fig. 26d).

## Summary and Perspectives

Generally speaking, ZIBs have achieved rapid development due to their high specific capacity, safety, environmental friendliness, and low cost. As an efficient ESS, the aqueous ZIBs are expected to dominate the future energy storage market. Unfortunately, the problem of anode stability in mild aqueous ZIBs has not been well resolved. It has become an insurmountable stumbling block for the commercialization and large-scale application of mild aqueous ZIBs. Hence, in this review, three critical issues that plague the performance of mild aqueous ZIB anodes are analyzed in detail, including dendrite growth caused by the inherently limited diffusion of Zn^2+^ ions, hydrogen evolution, and corrosion induced by water splitting. Among them, dendrites and hydrogen evolutions are the most direct damage to battery performance and life, especially for those devices with large capacity. The anode interface is prone to rapid deterioration, thus resulting in battery short circuits or bulging. Different from the above two intuitive effects, the corrosion pits and by-products displayed at the corrosion site may not directly damage the battery but gradually reduce the battery performance since excessive Zn metal has a certain tolerance to anode corrosion. Importantly, these anode issues are closely interconnected and mutually reinforcing. Although there is a certain theoretical basis for the formation mechanism and influencing factors, the insightful understandings are far from enough, and controversies on some basic issues are still unable to reach consensus. For example, a large current density is more likely to cause Zn dendrites than a small one. Still, some researchers insist that a large current density is more conducive to dense Zn deposition and inhibits Zn dendrites. Subsequently, in the anode optimization part, the current relevant strategies to improve the anode performance were summarized combined with recent related reports. From the theoretical analysis perspective, each strategy’s modification mechanism is explained in detail, involving interface modification, structural anode, alloying anode, intercalation anode, liquid electrolyte, non-liquid electrolyte, separator design, and other strategies. To better track the performance progress of ZIBs, the performance list of ZIBs related to Zn anode research are also summarized (Table S1 in the Supporting Information).

Through detailed analysis of the latest developments, it can be concluded that although the anode interface stability problem has made great progress, there is still much room for improvement. Therefore, some suggestions are proposed here:

1. Deepen the research of fundamental mechanisms. Currently, the explanation of mechanisms that expound the influence of various strategies on the Zn deposition behavior in mild solutions is mainly based on ectopic analysis and theoretical simulations without enough convincing evidence. The interface reaction between the anode and the electrolyte remains ambiguous, and more attention is paid to the deposition process, but the stripping process is frequently ignored. Hence, it is necessary to conduct in-depth and systematic research on the Zn deposition and stripping mechanism in mild solutions from the molecular and atomic levels. Correspondingly, more comprehensive characterization techniques are required to explore the fundamental mechanisms, especially the direct observation that can obtain convincing first-hand data simply, precisely, and quickly. For example, in situ visualization characterization techniques (such as X-ray phase-contrast imaging, in situ SEM, in situ XRD, and in situ optical microscopy) are constantly evolving, which can be combined with phase-field simulation to analyze complex electrochemical behaviors. Also, significantly, the theoretical calculation (DFT calculation and other computational models) should be developed to predict and verify Zn deposition/stripping tendency on the anode and provide clear theoretical support for nucleation and growth at the interface.
2. Formulate and improve quantify and test evaluation standards. Attributable to the failure of some critical parameters to be reasonably quantified, such as Zn reversibility. The current evaluation standard of anode reversibility is based on its lifespan and stability. Nevertheless, it ignores the irreversible process that excess Zn can make up for the capacity loss caused by Zn death or corrosion, which is at the cost of reducing the battery’s energy density. Besides, most reports ignore the detailed quantification of H_2_ evolution regardless of mild electrolytes that are more likely to cause HER than alkaline electrolytes, which increases the difficulty of analyzing the mechanism of capacity loss and battery failure. Besides, almost all reports claimed that they had constructed a perfect Zn anode and obtained enhanced battery performance accordingly. But their results are based on different test protocols such as the amount of electrolyte, test device, test temperature, current density, and depth of discharge (DOD). These test conditions are challenging to be unified, so that the experimental data results lack comparability to a certain extent. Therefore, utilizing multiple methods to form and improve quantify and test evaluation standards is beneficial to developing Zn anodes.
3. Combine multiple strategies. Given that the anode interface problems are interconnected, a combination of strategies to solve various issues simultaneously can make up for the limited improvement in the anode. For example, some electrolyte additives can not only coordinate with Zn^2+^ ions to destroy the solvation structure, but can also be adsorbed on the metal surface to shield the direct contact of water and limit Zn nucleation and growth area. Combining structured anodes with additives or introducing additives into the gel electrolyte can achieve a stable anode interface. In addition, by introducing metal or metal oxide particles into the modified layer or structured anode, the composite anode can be constructed to strengthen the regulation of Zn deposition.
4. Develop multifunctional separators. Since separators are connected to all the components of ZIBs, suitable separators can benefit the performance of both cathode and anode at the same time. However, separators commonly used in laboratories (glass fiber or cellulose separators) are challenging to maintain the long-term stability of anodes because they have poor mechanical strength and uneven ion transport channels. At present, there is little research on separators. Given that anode and separator are closely connected in the battery configuration, some anode interface modification strategies can be applied to the separator, such as compounding separators with some organic molecules with polar groups to obtain zincophilicity or constructing negative surface charges to inhibit anion transport and guide uniform cation migration.
5. Focus on comprehensive cost and benefits. Commercial batteries emphasize cost and energy density. Especially for the former, Zn content should be reasonably constrained to ensure good cycling stability. However, to deliberately pursue the excellent electrochemical performance of the battery, some aqueous ZIBs contain excessive Zn (most Zn does not participate in the electrochemical reaction) or use high-concentration electrolyte strategies, which will dramatically increase the cost and fundamentally reduce the battery energy density. Applying anodes with a high specific surface area, such as Zn powder, is a promising method to increase Zn utilization and Zn content. Moreover, compared with other energy storage devices, low cost, high safety, and environmental friendliness are the basic characteristics of aqueous ZIBs, essential requirements for long-term development. But considerable research places too much emphasis on dendrite-free and side reaction anodes but ignores these factors. Instead, they adopt expensive and harmful raw materials or complex technical routes that are difficult to produce on a large scale. Based on this status quo, it is recommended that the cost and benefit are comprehensively evaluated in designing reversible Zn anodes.

## Supplementary Information

Below is the link to the electronic supplementary material.

Supplementary file1 (PDF 249 kb)
