# Supplementary material for: Zinc Anode for Mild Aqueous Zinc-Ion Batteries: Challenges, Strategies, and Perspectives
Source: Nanomicro Lett. 2022 Jan 3;14:42. doi: 10.1007/s40820-021-00782-5 (PMC8724388; doi:10.1007/s40820-021-00782-5)
Supplement: Supplementary file 1 — Supplementary file1 (PDF 249 kb) [file 40820_2021_782_MOESM1_ESM.pdf]

Supporting Information for

Zinc Anode for Mild Aqueous Zinc-Ion Batteries: Challenges, Strategies, and Perspectives

Jinzhang Yang[1], Bosi Yin[1], Ying Sun[1], Hongge Pan[2, 3], Wenping Sun[3], Baohua Jia[4], Siwen Zhang[1, *], Tianyi Ma[4, *]

[1]Institute of Clean Energy Chemistry, Key Laboratory for Green Synthesis and Preparative Chemistry of Advanced Materials of Liaoning Province, College of Chemistry, Liaoning University, Shenyang 110036, P. R. China

[2]Institute of Science and Technology for New Energy, Xi'an Technological University, Xi'an 710021, P. R. China

[3]School of Materials Science and Engineering, State Key Laboratory of Clean Energy Utilization, Zhejiang University, Hangzhou 310027, P. R. China

[4]Centre for Translational Atomaterials, Swinburne University of Technology, Hawthorn, VIC 3122, Australia

*Corresponding authors. E-mail: zhangsiwen@lnu.edu.cn (S. Zhang); tianyima@swin.edu.au (T. Ma)

Supplementary Table

Table S1 Summary of the electrochemical performance for some mild aqueous ZIBs

Anode	Electrolyte	Current density (mA cm^{-2})	Area capacity (mAh cm^{-2})	Performance of pristine symmetric batteries		Performance of improved symmetric batteries		Refs.
				Voltage hysteresis (mV)	Lifespan (h)	Voltage hysteresis (mV)	Lifespan (h)	
Nafion-Zn-X layer	2 M $ZnSO_4$	2	0.5	90	71 cycles	50	1000 cycles	[S1]
HsGDY layer	2 M $ZnSO_4$	2	0.1	~228	63	~120	>2400	[S2]
Nanoporous $CaCO_3$	3 M $ZnSO_4$ + 0.1 M $MnSO_4$	0.25	0.05	230	55	80	836	[S3]
Cyanoacrylate layer	2 M $ZnSO_4$	2	1	~91.3	~60	~111.2	400	[S4]
3D ZnF_2 layer	2 M $ZnSO_4$	1	1.0	128	#	~71.5	800	[S5]
Polyimide layer	2 M $ZnSO_4$	4	2	~100	105	25	300	[S6]
Zn@ZnO-3D	3 M $ZnSO_4$ + 0.1 M $MnSO_4$	5	1.25	#	#	43	500	[S7]
Graphite	2 M $ZnSO_4$	0.1	0.1	#	~70	~28	200	[S8]
3D carbon nanotube	2 M $ZnSO_4$	2	2	#	50	27	200	[S9]
MXene layer	2 M $ZnSO_4$	0.2	0.2	~125	167	47	800	[S10]
ZIF-8 layer	2 M $ZnSO_4$	2	1	~60	130	~58	1200	[S11]
Au nanoparticles	3 M $ZnSO_4$	0.25	0.05	~80	92	~60	2000	[S12]

Sn coating	2 M $ZnSO_4$	1	0.5	~130	120	50	500	[S13]
Cu interfacial layer	3 M $ZnSO_4$ + 0.1 M $MnSO_4$	5	2	~75	84	31.5	1500	[S14]
Ag interfacial layer	3 M $ZnSO_4$ + 0.1 M $MnSO_4$	5	2	~75	84	28.3	448	[S15]
liquid Ga-In alloy coating	3 M $ZnSO_4$	0.25	0.05	72	80	24	2100	[S16]
liquid Ga-In-Zn alloy layer	2 M $ZnSO_4$	1	0.5	#	100	~20	500	[S17]
In microparticles	2 M $ZnSO_4$	0.2	0.2	261	~150	54	1500	[S18]
β-PVDF	2 M $ZnSO_4$	0.25	0.05	~165	350	80	2000	[S19]
AEC coating	2 M $ZnSO_4$	0.885	0.885	<50	~450	<50	2000	[S20]
Alucone	3 M $Zn(SO_3CF_3)_2$	3	1	84.3	70	110.3	780	[S21]
Cu mesh	1 M $ZnSO_4$	1	1	>35	60	20	340	[S22]
3D Ni–Zn anode	2 M $ZnSO_4$	5	2	160	20	80	200	[S23]
3D nanoporous Zn	2 M $Zn(CF_3SO_3)_2$	0.5	0.1	~100	220	60	1400	[S24]
Copper foam	2 M $ZnSO_4$ + 0.1 M $MnSO_4$	2	1	#	~145	~40	150	[S25]
3D porous copper skeleton	2 M $ZnSO_4$	0.5	0.5	100	110	40	350	[S26]
3D ridge structure Zn	3 M $ZnSO_4$	1	0.5	72	39	20	200	[S27]
3D ZnP/CF	2 M $ZnSO_4$	1	1	482	170	25.1	3000	[S28]
Carbon fiber	3 M $Zn(CF_3SO_3)_2$	0.25	0.125	80	110	46	200	[S29]
Graphite fiber	0.5 M $NaSO_4$ + 0.05 M $ZnSO_4$	1	1	~100	50 cycles	~50	350 cycles	[S30]
ZIF-8-500 layer	2 M $ZnSO_4$	2	1	#	#	#	>200 cycles	[S31]
$Ti_3C_2T_x$ MXene@Zn paper	2 M $ZnSO_4$	1	1	>109	~78	~83	300	[S32]
Cu–Zn nanoalloy	3 M $ZnSO_4$	1	0.5	>400	#	~46	1500	[S33]
Eutectic $Zn_{88}Al_{12}$ alloy	2 M $ZnSO_4$	0.5	0.5	~101	~50	~20	2000	[S34]
3D Zn-Mn alloy	2 M $ZnSO_4$ in seawater	80	16	~600	<30	~300	760	[S35]
Zn	$ZnSO_4$ + glucose	1	1	#	300	#	2000	[S36]
Zn	$ZnSO_4$ + glycerol	2	6	#	100	#	900	[S37]
Zn	$ZnSO_4$ + amino acid	5	4	#	70	#	2200	[S38]
Zn	$ZnSO_4$ + ZnF_2	1	1	165	~80	124	600	[S39]
Zn	$ZnSO_4$ + KPF_6	2	4	#	95	#	1200	[S40]
Zn	ZPSAM	2	0.5	#	55	#	1000	[S41]
Zn	PAMPSZn	1	1	#	139	100	4500	[S42]
Zn	PVHF/MXene -g-$PMA^{0.05}$	0.2	0.2	#	#	#	1200	[S43]

Supplementary References

[S1] Y. Cui, Q. Zhao, X. Wu, X. Chen, J. Yang et al., An interface-bridged organic–inorganic layer that suppresses dendrite formation and side reactions for ultra-long-life aqueous zinc metal anodes. Angew. Chem. Int. Ed. **59**(38), 16594–16601 (2020). https://doi.org/10.1002/anie.202005472

[S2] Q. Yang, Y. Guo, B. Yan, C. Wang, Z. Liu et al., Hydrogen-substituted graphdiyne ion tunnels directing concentration redistribution for commercial-grade dendrite-free zinc anodes. Adv. Mater. **32**(25), 2001755 (2020). https://doi.org/10.1002/adma.202001755

[S3] L. Kang, M. Cui, F. Jiang, Y. Gao, H. Luo et al., Nanoporous $CaCO_3$ coatings enabled uniform Zn stripping/pating for long-life zinc rechargeable aqueous batteries. Adv. Energy Mater. **8**(25), 1801090 (2018). https://doi.org/10.1002/aenm.201801090

[S4] Z. Cao, X. Zhu, D. Xu, P. Dong, M.O.L. Chee et al., Eliminating Zn dendrites by commercial cyanoacrylate adhesive for zinc ion battery. Energy Storage Mater. **36**, 132–138 (2021). https://doi.org/10.1016/j.ensm.2020.12.022

[S5] Y. Yang, C. Liu, Z. Lv, H. Yang, Y. Zhang et al., Synergistic manipulation of Zn^{2+} ion flux and desolvation effect enabled by anodic growth of a 3D ZnF_2 matrix for long-lifespan and dendrite-Free Zn metal anodes. Adv. Mater. **33**(11), 2007388 (2021). https://doi.org/10.1002/adma.202007388

[S6] M. Zhu, J. Hu, Q. Lu, H. Dong, D.D. Karnaushenko et al., A patternable and in situ formed polymeric zinc blanket for a reversible zinc anode in a skin-mountable microbattery. Adv. Mater. **33**(8), 2007497 (2021). https://doi.org/10.1002/adma.202007497

[S7] X. Xie, S. Liang, J. Gao, S. Guo, J. Guo et al., Manipulating the ion-transfer kinetics and interface stability for high-performance zinc metal anodes. Energy Environ. Sci. **13**(2), 503–510 (2020). https://doi.org/10.1039/c9ee03545a

[S8] Z. Li, L. Wu, S. Dong, T. Xu, S. Li et al., Pencil drawing stable interface for reversible and durable aqueous zinc-ion batteries. Adv. Funct. Mater. **31**(4), 2006495 (2021). https://doi.org/10.1002/adfm.202006495

[S9] L. Dong, W. Yang, W. Yang, H. Tian, Y. Huang et al., Flexible and conductive scaffold-stabilized zinc metal anodes for ultralong-life zinc-ion batteries and zinc-ion hybrid capacitors. Chem. Eng. J. **384**, 123355 (2020). https://doi.org/10.1016/j.cej.2019.123355

[S10] N. Zhang, S. Huang, Z. Yuan, J. Zhu, Z. Zhao et al., Direct self-assembly of MXene on Zn anodes for dendrite-free aqueous zinc-ion batteries. Angew. Chem. Int. Ed. **60**(6), 2861–2865 (2021). https://doi.org/10.1002/anie.202012322

[S11] R. Yuksel, O. Buyukcakir, W.K. Seong, R.S. Ruoff, Metal-organic framework integrated anodes for aqueous zinc-ion batteries. Adv. Energy Mater. **10**(16), 1904215 (2020). https://doi.org/10.1002/aenm.201904215

[S12] M. Cui, Y. Xiao, L. Kang, W. Du, Y. Gao et al., Quasi-isolated Au particles as heterogeneous seeds to guide uniform Zn deposition for aqueous zinc-ion batteries. ACS Appl. Energy Mater. **2**(9), 6490–6496 (2019). https://doi.org/10.1021/acsaem.9b01063

[S13] Y. Yin, S. Wang, Q. Zhang, Y. Song, N. Chang et al., Dendrite-free zinc deposition induced by tin-modified multifunctional 3D host for stable zinc-based flow battery. Adv. Mater. **32**(6), 1906803 (2020). https://doi.org/10.1002/adma.201906803

[S14] Y. Zhang, G. Wang, F. Yu, G. Xu, Z. Li et al., Highly reversible and dendrite-free Zn electrodeposition enabled by a thin metallic interfacial layer in aqueous batteries. Chem. Eng. J. **416**, 128062 (2021). https://doi.org/10.1016/j.cej.2020.128062

[S15] Y. Wang, Y. Chen, W. Liu, X. Ni, P. Qing et al., Uniform and dendrite-free zinc deposition enabled by in situ formed AgZ_3 for the zinc metal anode. J. Mater. Chem. A **9**(13), 8452–8461 (2021). https://doi.org/10.1039/D0TA12177K

[S16] C. Liu, Z. Luo, W. Deng, W. Wei, L. Chen et al., Liquid alloy interlayer for aqueous zinc-ion battery. ACS Energy Lett. **6**(2), 675–683 (2021). https://doi.org/10.1021/acsenergylett.0c02569

[S17] H. Jia, Z. Wang, M. Dirican, S. Qiu, C.Y. Chan et al., A liquid metal assisted dendrite-free anode for high-performance Zn-ion batteries. J. Mater. Chem. A **9**(9), 5597–5605 (2021). https://doi.org/10.1039/d0ta11828a

[S18] D. Han, S. Wu, S. Zhang, Y. Deng, C. Cui et al., A corrosion-resistant and dendrite-free Zinc metal anode in aqueous systems. Small **16**(29), 2001736 (2020). https://doi.org/10.1002/smll.202001736

[S19] L.T. Hieu, S. So, I.T. Kim, J. Hur, Zn anode with flexible β-PVDF coating for aqueous Zn-ion batteries with long cycle life. Chem. Eng. J. **411**, 128584 (2021). https://doi.org/10.1016/j.cej.2021.128584

[S20] R. Zhao, Y. Yang, G. Liu, R. Zhu, J. Huang et al., Redirected Zn electrodeposition by an anti-corrosion elastic constraint for highly reversible Zn anodes. Adv. Funct. Mater. **31**(2), 2001867 (2021). https://doi.org/10.1002/adfm.202001867

[S21] H. He, J. Liu, Suppressing Zn dendrite growth by molecular layer deposition to enable long-life and deeply rechargeable aqueous Zn anodes. J. Mater. Chem. A **8**(42), 22100–22110 (2020). https://doi.org/10.1039/d0ta07232j

[S22] Q. Zhang, J. Luan, X. Huang, L. Zhu, Y. Tang et al., Simultaneously regulating the ion distribution and electric field to achieve dendrite-free Zn anode. Small **16**(35), 2000929 (2020). https://doi.org/10.1002/smll.202000929

[S23] G. Zhang, X. Zhang, H. Liu, J. Li, Y. Chen et al., 3D-printed multi-channel metal lattices enabling localized electric-field redistribution for dendrite-free aqueous Zn ion batteries. Adv. Energy Mater. **11**(19), 2003927 (2021). https://doi.org/10.1002/aenm.202003927

[S24] W. Guo, Z. Cong, Z. Guo, C. Chang, X. Liang et al., Dendrite-free Zn anode with dual channel 3D porous frameworks for rechargeable Zn batteries. Energy Storage Mater. **30**, 104–112 (2020). https://doi.org/10.1016/j.ensm.2020.04.038

[S25] C. Li, X. Shi, S. Liang, X. Ma, M. Han et al., Spatially homogeneous copper foam as surface dendrite-free host for zinc metal anode. Chem. Eng. J. **379**, 122248 (2020). https://doi.org/10.1016/j.cej.2019.122248

[S26] Z. Kang, C. Wu, L. Dong, W. Liu, J. Mou et al., 3D porous copper skeleton supported zinc anode toward high capacity and long cycle life zinc ion batteries. ACS Sustain. Chem. Eng. **7**(3), 3364–3371 (2019). https://doi.org/10.1021/acssuschemeng.8b05568

[S27] J. Wang, Z. Cai, R. Xiao, Y. Ou, R. Zhan et al., A chemically polished zinc metal electrode with a ridge-like structure for cycle-stable aqueous batteries. ACS Appl.

Mater. Interfaces **12**(20), 23028–23034 (2020). https://doi.org/10.1021/acsami.0c05661

[S28] Y. Du, X. Chi, J. Huang, Q. Qiu, Y. Liu, Long lifespan and high-rate Zn anode boosted by 3D porous structure and conducting network. J. Power Sources. **479**, 228808 (2020). https://doi.org/10.1016/j.jpowsour.2020.228808

[S29] S. Zhai, N. Wang, X. Tan, K. Jiang, Z. Quan et al., Interface-engineered dendrite-free anode and ultraconductive cathode for durable and high-rate fiber Zn dual-ion microbattery. Adv. Funct. Mater. **31**(13), 2008894 (2021). https://doi.org/10.1002/adfm.202008894

[S30] L.P. Wang, N.W. Li, T.S. Wang, Y.X. Yin, Y.G. Guo et al., Conductive graphite fiber as a stable host for zinc metal anodes. Electrochim. Acta **244**, 172–177 (2017). https://doi.org/10.1016/j.electacta.2017.05.072

[S31] Z. Wang, J. Huang, Z. Guo, X. Dong, Y. Liu et al., A metal-organic framework host for highly reversible dendrite-free zinc metal anodes. Joule **3**(5), 1289–1300 (2019). https://doi.org/10.1016/j.joule.2019.02.012

[S32] Y. Tian, Y. An, C. Wei, B. Xi, S. Xiong et al., Flexible and free-standing $Ti_3C_2T_x$ MXene@Zn paper for dendrite-free aqueous zinc metal batteries and nonaqueous lithium metal batteries. ACS Nano **13**(10), 11676–11685 (2019). https://doi.org/10.1021/acsnano.9b05599

[S33] Z. Cai, Y. Ou, J. Wang, R. Xiao, L. Fu et al., Chemically resistant Cu–Zn/Zn composite anode for long cycling aqueous batteries. Energy Storage Mater. **27**, 205–211 (2020). https://doi.org/10.1016/j.ensm.2020.01.032

[S34] S.B. Wang, Q. Ran, R.Q. Yao, H. Shi, Z. Wen et al., Lamella-nanostructured eutectic zinc–aluminum alloys as reversible and dendrite-free anodes for aqueous rechargeable batteries. Nat. Commun. **11**, 1634 (2020). https://doi.org/10.1038/s41467-020-15478-4

[S35] H. Tian, Z. Li, G. Feng, Z. Yang, D. Fox et al., Stable, high-performance, dendrite-free, seawater-based aqueous batteries. Nat. Commun. **12**, 237 (2021). https://doi.org/10.1038/s41467-020-20334-6

[S36] P. Sun, L. Ma, W. Zhou, M. Qiu, Z. Wang et al., Simultaneous regulation on solvation shell and electrode interface for dendrite-free Zn ion batteries achieved by a low-cost glucose additive. Angew. Chem. Int. Ed. **60**(33), 18247–18255 (2021). https://doi.org/10.1002/anie.202105756

[S37] Y. Zhang, M. Zhu, K. Wu, F. Yu, G. Wang et al., An in-depth insight of a highly reversible and dendrite-free Zn metal anode in an hybrid electrolyte. J. Mater. Chem. A **9**(7), 4253–4261 (2021). https://doi.org/10.1039/d0ta11668h

[S38] H. Lu, X. Zhang, M. Luo, K. Cao, Y. Lu et al., Amino acid-induced interface charge engineering enables highly reversible Zn anode. Adv. Funct. Mater. **31**(45), 2103514 (2021). https://doi.org/10.1002/adfm.202103514

[S39] D. Li, L. Cao, T. Deng, S. Liu, C. Wang, Design of a solid electrolyte interphase for aqueous Zn batteries. Angew. Chem. Int. Ed. **60**(23), 13035–13041 (2021). https://doi.org/10.1002/anie.202103390

[S40] Y. Chu, S. Zhang, S. Wu, Z. Hu, G. Cui et al., In situ built interphase with high interface energy and fast kinetics for high performance Zn metal anodes. Energy Environ. Sci. **14**(6), 3609–3620 (2021). https://doi.org/10.1039/D1EE00308A

[S41] L. Ma, S. Chen, N. Li, Z. Liu, Z. Tang et al., Hydrogen-free and dendrite-free all-solid-state Zn-ion batteries. Adv. Mater. **32**(14), 1908121 (2020). https://doi.org/10.1002/adma.201908121

[S42] J. Cong, X. Shen, Z. Wen, X. Wang, L. Peng et al., Ultra-stable and highly reversible aqueous zinc metal anodes with high preferred orientation deposition achieved by a polyanionic hydrogel electrolyte. Energy Storage Mater. **35**, 586–594 (2021). https://doi.org/10.1016/j.ensm.2020.11.041

[S43] Z. Chen, X. Li, D. Wang, Q. Yang, L. Ma et al., Grafted MXene/polymer electrolyte for high performance solid zinc batteries with enhanced shelf life at low/high temperatures. Energy Environ. Sci. **14**(6), 3492–3501 (2021). https://doi.org/10.1039/D1EE00409C
